# A pre-existing *Toxoplasma gondii* infection exacerbates the pathophysiological response and extent of brain damage after traumatic brain injury in mice

**DOI:** 10.1186/s12974-024-03014-w

**Published:** 2024-01-09

**Authors:** Tamara L. Baker, David K. Wright, Alessandro D. Uboldi, Christopher J. Tonkin, Anh Vo, Trevor Wilson, Stuart J. McDonald, Richelle Mychasiuk, Bridgette D. Semple, Mujun Sun, Sandy R. Shultz

**Affiliations:** 1 Department of Neuroscience, Central Clinical School, Monash University, 6th Floor, The Alfred Centre, 99 Commercial Road, Melbourne, VIC 3004 Australia; 2 Division of Infectious Disease and Immune Defense, , The Walter and Eliza Hall Institute of Medical Research, Parkville, VIC Australia; 3 Department of Medical Biology, The University of Melbourne, Melbourne, VIC 3010 Australia; 4 Monash Health Translation Precinct, Monash University, Melbourne, VIC Australia; 5 Health Sciences, Vancouver Island University, Nanaimo, BC Canada

**Keywords:** Neuroinflammation, Immune response, Oxidative stress, Excitotoxicity, Females, Sex, MRI, Behavior

## Abstract

Traumatic brain injury (TBI) is a key contributor to global morbidity that lacks effective treatments. Microbial infections are common in TBI patients, and their presence could modify the physiological response to TBI. It is estimated that one-third of the human population is incurably infected with the feline-borne parasite, *Toxoplasma gondii,* which can invade the central nervous system and result in chronic low-grade neuroinflammation, oxidative stress, and excitotoxicity—all of which are also important pathophysiological processes in TBI. Considering the large number of TBI patients that have a pre-existing *T. gondii* infection prior to injury, and the potential mechanistic synergies between the conditions, this study investigated how a pre-existing *T. gondii* infection modified TBI outcomes across acute, sub-acute and chronic recovery in male and female mice. Gene expression analysis of brain tissue found that neuroinflammation and immune cell markers were amplified in the combined *T. gondii* + TBI setting in both males and females as early as 2-h post-injury. Glutamatergic, neurotoxic, and oxidative stress markers were altered in a sex-specific manner in *T. gondii* + TBI mice. Structural MRI found that male, but not female, *T. gondii* + TBI mice had a significantly larger lesion size compared to their uninfected counterparts at 18-weeks post-injury. Similarly, diffusion MRI revealed that *T. gondii* + TBI mice had exacerbated white matter tract abnormalities, particularly in male mice. These novel findings indicate that a pre-existing *T. gondii* infection affects the pathophysiological aftermath of TBI in a sex-dependent manner, and may be an important modifier to consider in the care and prognostication of TBI patients.

**Supplementary Information:**

The online version contains supplementary material available at 10.1186/s12974-024-03014-w.

## Background

Traumatic brain injury (TBI) is a global health concern, and the estimated annual incidence exceeds 27 million worldwide [1, 2]. TBI survivors often experience debilitating motor, cognitive, social, and emotional deficits that can persist chronically [3–6]. These long-term functional consequences are believed to be underpinned by neuropathology resulting from both primary (i.e., brain damage caused by mechanical forces at the time of impact) and secondary injury mechanisms (i.e., delayed and/or evolving processes triggered by the primary insult), including influential secondary pathways such as neuroinflammation, excitotoxicity, and oxidative stress [7–10].

The vast majority of preclinical TBI studies have applied isolated TBI models in male subjects [11]. Although these studies have made important contributions to the field, the translation of these findings to humans is inherently limited because they do not account for fundamental variations, concurrent factors, and co-morbid conditions that occur in the clinical setting (e.g., biological sex, peripheral injuries, heart conditions, diabetes, stroke, respiratory illness, infections) that may modify the physiological response to TBI and contribute to the heterogeneity that occurs in patients [12–18]. For example, infections are common in TBI patients, can impact TBI secondary pathways (e.g., immune response), and have the potential to influence outcomes [19, 20]. Despite the important implications that an infection could have on patient care, few preclinical TBI studies have investigated clinically relevant pathogens. Furthermore, existing studies have only considered infection as a ‘secondary hit’ (i.e., infection that occurs after TBI) [21–31]. As such, there is a need to study holistic infection/pathogen models and understand how pre-existing infections may influence TBI outcomes.

Approximately one-third of the human population is chronically and incurably infected with *Toxoplasma gondii,* an obligate intracellular parasite belonging to the Apicomplexa phylum [34]. *T. gondii* exclusively sexually reproduces in members of the Felidae family, such as domestic cats; however, most mammals can become infected [32]. *T. gondii* is most often transmitted via ingestion of contaminated food, water and tainted/infected animal products, but it can also spread via blood transfusion, organ transplantation, or vertical transmission [33–36]. *T. gondii* can hijack immune cells, disseminate throughout a host, and invade numerous cell types to replicate and survive [37–39]. *T. gondii* preferentially enters the central nervous system where it can occupy neurons, differentiate into an encysted and slow replicating form, and resist immune clearance [38–40]. Most clinical cases of chronic *T. gondii* infection occur with type II strains such as Prugniaud (Pru), and chronic infection is characterized by sustained sub-clinical neuroinflammation, increased oxidative stress, and disrupted glutamate homeostasis [41–43]. All of these pathological processes are also prominent features of secondary injury after a TBI [44, 45].

Assuming *T. gondii* infection rates are somewhat similar in individuals who sustain a TBI and the general public, a large number of TBI patients would therefore have a pre-existing *T. gondii* infection at the time of their injury. As such, here we investigated how a pre-existing *T. gondii* infection modifies TBI outcomes in male and female mice at acute, sub-acute and chronic recovery timepoints. Since each isolated condition can promote neuroinflammatory, oxidative stress, and excitotoxic processes that can contribute to long-term pathological and behavioral alterations, we hypothesized that in a combined *T. gondii* + TBI setting the pathobiological processes would be exacerbated and result in worse long-term outcomes.

## Materials and methods

### Animals

A total of 308 C57BL/6 J (Jax) (157 male, 151 female) mice were obtained from the Alfred Medical Research and Education Precinct (AMREP) Animal Services (Melbourne, Australia). Mice were housed in ventilated Optimice^®^ cages under a 12-h light/dark cycle, with access to food and water ad libitum. Mice were group-housed (3–6 mice/cage/sex) with the exception of the long-recovery groups being individually housed beginning the day prior to social testing. All procedures were approved by the Alfred Animal Ethics Committee (#E/2005/2020/M) and conducted in accordance with the guidelines of the Australian Code of Practice for the Care and Use of Animals for Scientific Purposes by the Australian National Health and Medical Research Council.

### Experimental design

**Fig. 1:**
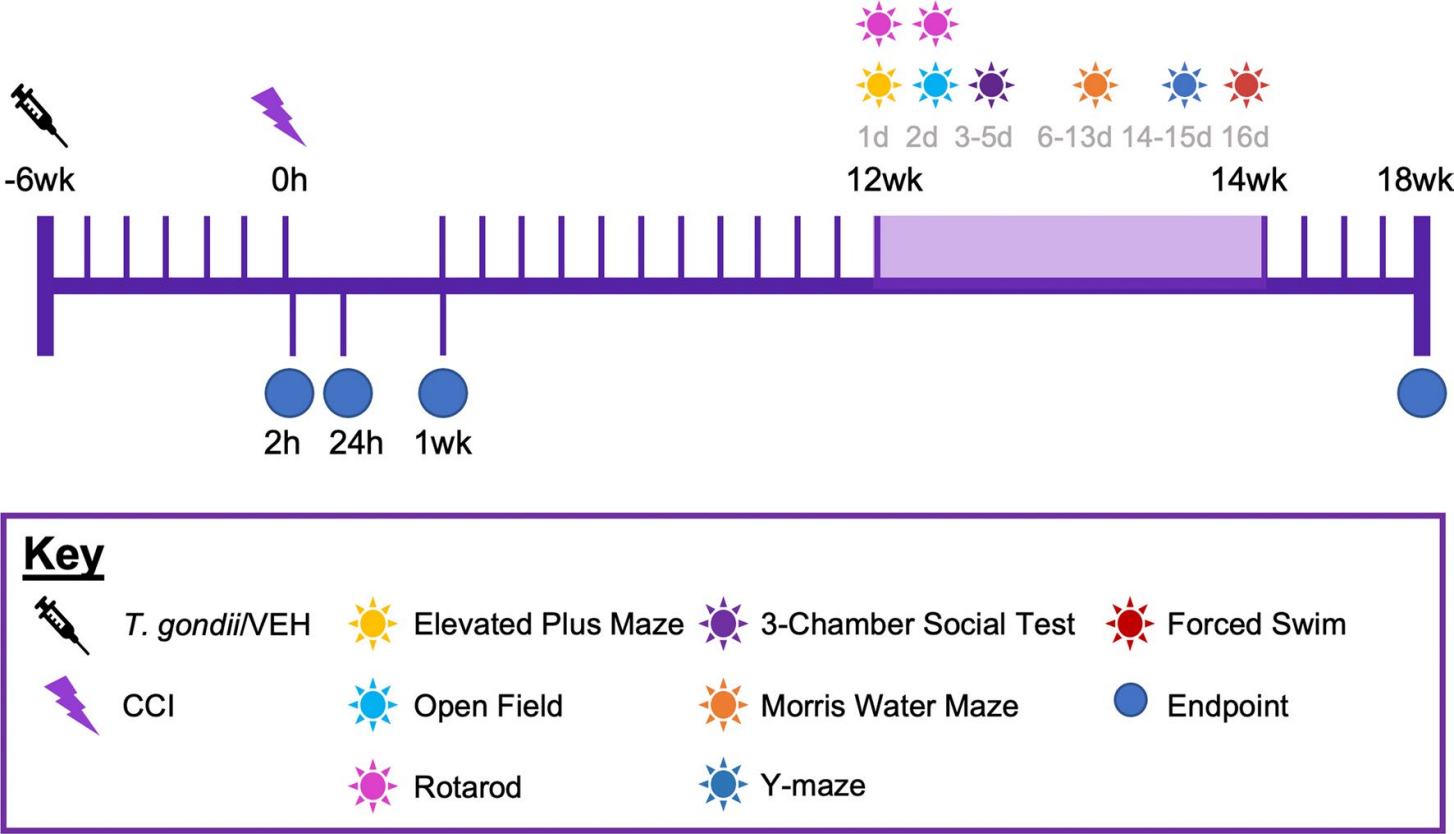
Study design. All mice were injected with either *Toxoplasma gondii* (*T. gondii*) or vehicle (VEH) and given six-weeks for a chronic infection to establish. At this point, mice received either a controlled cortical impact (CCI) or sham injury. From this point, mice were allocated to either acute endpoints of 2-h or 24-h, or a sub-acute endpoint of 1-week recovery. Alternatively, mice were allocated to a long-term (i.e., 18-week) recovery, during which all mice underwent neurobehavioral testing beginning at 12-weeks post-injury

Mice were randomly allocated to receive either a single intraperitoneal (i.p.) injection of *T. gondii* tachyzoites or vehicle at six-weeks of age (Fig. 1). This age was chosen because *T. gondii* infection is common in children and adolescence (~ 10 to 25% infection rate) [46–50]. Furthermore, TBI is common in young-adults and the CCI model has been well-validated in this age group [51, 52]. Therefore, the infection was induced at six-weeks of age in order to administer the TBI/sham injury in young-adult mice (i.e., 12-weeks old) with a pre-existing chronic *T. gondii* infection. On days 5 to 10 after injection, all mice (including Vehicle-injected mice) received 100 µg/mL sulfadiazine sodium (Sigma Aldrich, Burlington, MA, USA) in their drinking water to assist recovery from the acute stage of infection. This therapy is often used to reduce mortality throughout the acute stage of infection and control tachyzoite proliferation during active toxoplasmosis, but it does not prevent infection from occurring [53, 54]. Of 164 mice (85 male, 79 female) infected with *T. gondii*, 20 (10 male, 10 female) did not recover from the acute stage of infection (12.2% total mortality). In addition, one *T. gondii* male mouse was excluded from this study, as no other *T. gondii* mice within this cohort recovered from the acute stage of infection. Six-weeks were allowed to pass for a chronic *T. gondii* infection to manifest [55], and at this point, mice received either a TBI that was induced via the controlled cortical impact (CCI) model or a sham injury. Therefore, this study was comprised of four experimental groups per sex: Vehicle + Sham; *T. gondii* + Sham; Vehicle + CCI; and *T. gondii* + CCI. Subsets of mice were euthanized at either 2-h (23 male, 24 female), 24-h (26 male, 25 female), or 1-week post injury (49 male, 46 female) to examine acute and sub-acute pathophysiology, and another subset received an 18-week recovery after TBI to examine chronic behavior and neuropathological differences (48 male, 46 female). Some mice (2 *T. gondii* + CCI males, 1 *T. gondii* + Sham female) died prematurely and as a result, 91 mice reached the 18-weeks post-injury endpoint. Investigators were blinded to group allocations throughout all experimentation and data analyses.

### Chronic *Toxoplasma gondii* infection

**Fig. 2:**
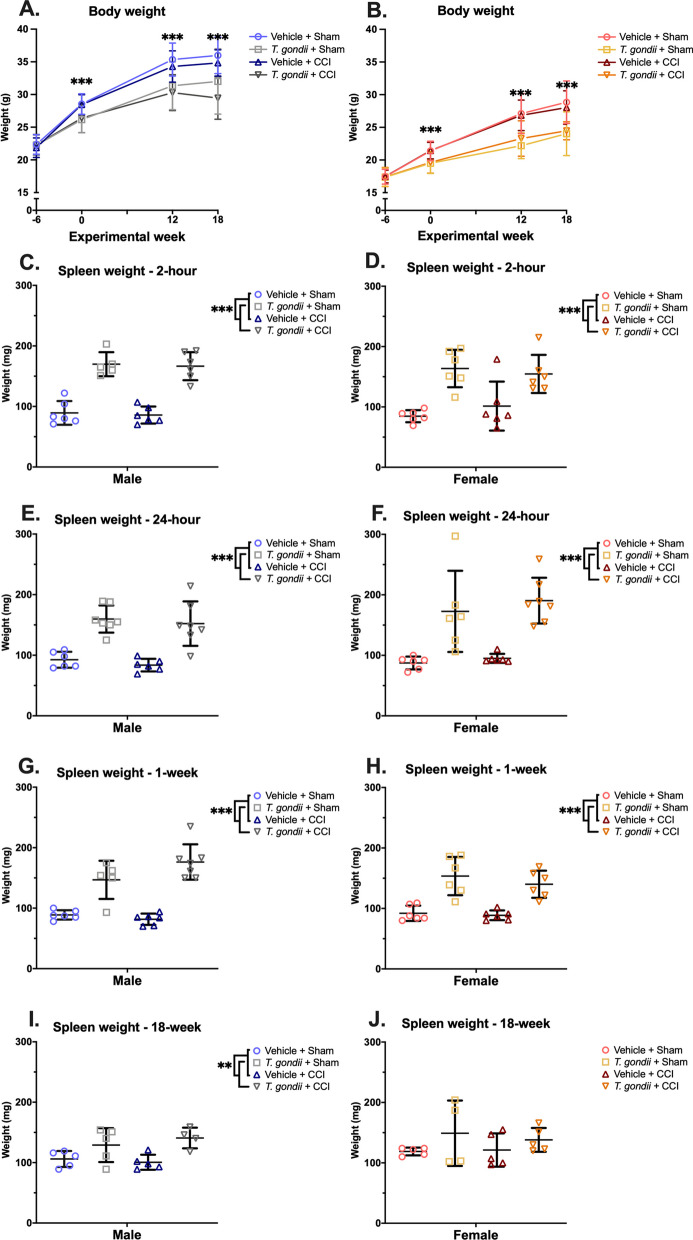
*T. gondii* infection leads to decreased body weight and an enlarged spleen in both sexes. In both males **(A)** and females **(B)**, *T. gondii* mice weighed less than Vehicle mice 6-weeks after infection (i.e., time of CCI/sham injury), as well as at 12- (i.e., at behavioral testing) and 18-weeks post-CCI/sham injury. Both male and female *T. gondii* mice had increased spleen weight compared to Vehicle mice at 2-h **(C, D)**, 24-h **(E, F)** and 1-week **(G, H)** post-injury. Only the male *T. gondii* mice had increased spleen weight compared to their Vehicle counterparts at 18-weeks post injury **(I, J)**. Data displayed as mean ± SD. **p ≤ 0.01. ***p ≤ 0.001

*T. gondii* (Pru:tdTomato) tachyzoites were maintained by passage on human foreskin fibroblasts and resuspended in Dulbecco’s phosphate buffered saline (DPBS) to a concentration of 50,000 *T. gondii* tachyzoites per 200 μL DPBS [55]. Mice that were randomly allocated to *T. gondii* groups received a single i.p. injection of 50,000 tachyzoites and were monitored for sickness behaviors across a 6-week period as previously described [55]. Mice allocated to vehicle groups received 200 μL DPBS i.p. only and were likewise monitored across a 6-week period. Throughout the study, body weights were monitored (Fig. 2A and B). By 6-weeks post-injection (i.e., experimental week 0), in both males and females, *T. gondii* mice weighed significantly less than vehicle mice, and this difference was sustained until the experimental endpoint.

### *T. gondii* IgG seropositivity

Infection status was verified via serologic testing with a colorimetric Mouse Toxoplasmosis Antibody (IgG) ELISA Kit (NBP2-60169, Novus Biologicals, Centennial, CO, USA) using serum samples collected from *T. gondii* and vehicle-injected mice at the time of euthanasia. As increased spleen weight is characteristic in chronic *T. gondii* infection [56], fresh spleen weights were also used as an indicator of infection (Fig. 2C–J).

### Controlled cortical impact

CCI was performed at 6-weeks post-injection (12-weeks old) using an electronic CCI device (Custom Design and Fabrication Inc., Sandston, VA) as previously described [57]. Anesthesia was induced by 4% isoflurane in 1 L/min oxygen and maintained at 1.5–2% isoflurane via nose cone for surgery. Following anesthesia induction and prior to surgery, all mice were subcutaneously (s.c.) administered analgesics; buprenorphine (0.05 mg/kg in saline; s.c. flank) and bupivacaine (1 mg/kg in saline, s.c. at incision site). Then, mice were stabilized in a stereotaxic frame and a midline incision was made to expose the skull. A ~ 3 mm diameter craniotomy was completed above the left parietal lobe with a microdrill (0.6 mm drill bit) at a position of − 1 mm posterior to Bregma, 1 mm lateral to the midline-sagittal suture, and 1 mm anterior to Lambda. An impact was delivered using moderate-to-severe injury parameters set at 4.5 m/s velocity, 1.71 mm depth and 150 ms dwell time. Sham injuries were performed as described above, without the delivery of the CCI.

### Tissue collection

Mice were euthanized 2-h, 24-h, 1-week or 18-weeks post-injury, with a single i.p. injection of sodium pentobarbitone (80 mg/kg; Lethabarb; Virbac, Australia). Whole blood was collected from the left ventricle in BD Vacutainer^®^ SST II 3.5 mL Advance tubes (Becton Dickinson, Franklin Lakes, NJ, USA; silica clot activator), coagulated at room temperature for 30 min, and then centrifuged (1500 g at room temperature for 10 min) for serum isolation. Next, fresh spleen tissue was collected and weighed, and fresh brain tissue, including the ipsilateral parietal cortex, was collected for gene expression analysis. All samples were frozen on dry ice and stored at − 80 °C prior to analysis.

A subset of mice at 18-weeks post-surgery underwent transcardial perfusion with ice-cold sterile saline (0.9% NaCl w/v) followed by 4% paraformaldehyde (PFA) to fix the brain tissue for ex-vivo magnetic resonance imaging (MRI). Brains were post-fixed in 4% PFA overnight, at 4 °C, washed twice in 1 × PBS, then transferred to 1 × PBS for storage at 4 °C prior to ex-vivo MRI.

### Gene expression analysis

Gene expression analysis was used to examine immune, oxidative stress, glutamate and angiogenic pathways, and blood–brain barrier (BBB) components in the injured cortex. Total RNA was isolated from 15 to 20 mg of ipsilateral parietal cortex tissue using a RNeasy^®^ Mini Kit (Qiagen). 200 ng of yielded RNA proceeded to cDNA synthesis using Quantabio qScript XLT cDNA SuperMix (Quantabio). Multiplex qPCR was performed with Fluidigm BioMark HD™. For each sample, 1.25 μL of the resulting cDNA was combined with 3.75 μL of Sample Pre-Mix (Life Technologies TaqMan^®^ PreAmp Master Mix and Pooled Taqman assays) and pre-amplified for 14 cycles. The reaction products were diluted 1:5 and loaded onto the Gene expression integrated fluidic circuit (IFC) according to Fluidigm^®^ IFC Standard Taqman Gene expression workflow. 49 TaqMan^®^ gene expression assays related to immune cells, the glutamate pathway, neuroinflammation, oxidative stress and BBB disruption, and 4 reference gene assays were used as detailed in Table 1. Cycle threshold (Ct) values were collected for analysis, using the 2^−ΔΔCt^ method. BBB disruption genes were only assessed at the 18-week recovery timepoint. *CD206* was not analyzed at 2-h post-injury due to minimal amplification across all samples at this timepoint. No significant effects were found on expression of the reference genes *YWHAZ*, *ACTB*, *GAPDH,* and *UBC*.

**Table 1:** Cell, glutamate pathway, neuroinflammatory, oxidative stress, blood–brain barrier (BBB) and reference genes analyzed via RT-PCR

	Gene name	Taqman assay ID		Gene name	Taqman assay ID
Reference			Neuroinflammation		
1	*YWHAZ*	Mm01722325_m1	26	*TSPO*	Mm00437828_m1
2	*ACTB*	Mm00607939_s1	27	*NLRP3*	Mm00840904_m1
3	*GAPDH*	Mm99999915_g1	28	*IL1β*	Mm00434228_m1
4	*UBC*	Mm02525934_g1	29	*IL2*	Mm00434256_m1
Cell markers			30	*IL6*	Mm00446190_m1
5	*GFAP*	Mm01253033_m1	31	*IL10*	Mm00439616_m1
6	*CD45*	Mm01293577_m1	32	*IL12p40*	Mm01288989_m1
7	*CD86*	Mm00444543_m1	33	*IL33*	Mm00505403_m1
8	*CD206*	Mm01329362_m1	34	*TNFα*	Mm99999068_m1
9	*TREM2*	Mm04209424_g1	35	*CSF1/ M-CSF*	Mm00432688_m1
10	*CCR5*	Mm01216171_m1	36	*CSF2/ GM-CSF*	Mm00438328_m1
11	*IBA1*	Mm00479862_g1	37	*CXCL10*	Mm00445235_m1
12	*TMEM119*	Mm00525305_m1	38	*IFNγ*	Mm00801778_m1
13	*GATA3*	Mm00484683_m1	39	*TGFβ/ TGIF1*	Mm01227699_m1
14	*CXCR3*	Mm00438259_m1	40	*ARG1*	Mm00475988_m1
15	*STAT1*	Mm01257286_m1	41	*CCL2*	Mm00441242_m1
16	*SOCS1*	Mm00782550_s1	42	*CCL4*	Mm00443111_m1
17	*FOXP3*	Mm00475162_m1	43	*CCL5*	Mm01302427_m1
18	*MAP2*	Mm00485231_m1	44	*CCL12*	Mm01617100_m1
Glutamate pathway			Oxidative stress		
19	*GLT1*	Mm01275814_m1	45	*IDO1*	Mm00492586_m1
20	*GLAST*	Mm01306917_g1	46	*NOS2*	Mm00440502_m1
21	*GAD1*	Mm04207432_g1	47	*CYBB*	Mm01287743_m1
22	*PHGDH*	Mm01623589_g1	BBB disruption/Vascular health		
23	*GLUL*	Mm00725701_s1	48	*OCLN*	Mm00500912_m1
24	*GRIN2B*	Mm00433820_m1	49	*TJP1/ZO1*	Mm00493699_m1
25	*PPARγ*	Mm01184322_m1	50	*ICAM1*	Mm00516023_m1
			51	*HIF1A*	Mm00468869_m1
			52	*VEGFA*	Mm00437306_m1
			53	*SYNAPTOPHYSIN*	Mm00436850_m1

### Ex-vivo MRI acquisition

Ex-vivo MRI was conducted on a subset of brains collected at 18-weeks post-injury that were not used for gene expression analysis (Male Vehicle + Sham = 7; Male *T. gondii* + Sham = 7; Male Vehicle + CCI = 7; Male *T. gondii* + CCI = 6; Female Vehicle + Sham = 7; Female *T. gondii* + Sham = 6; Female Vehicle + CCI = 7; Female *T. gondii* + CCI = 6).

Fixed brains were washed in PBS and positioned in fomblin (Solvay Solexis, USA) for imaging with a 9.4 T Bruker MRI. A 3D multi-gradient echo image was acquired to assess lesion size. Imaging parameters included: repetition time (TR) = 75 ms; echo times (TE) = 5, 10, 15, …, 50 ms; field of view (FOV) = 16.32 × 10.88 × 7.14 mm^3^; matrix size = 192 × 128 × 84; and resolution = 85 × 85 × 85 μm^3^. Images were reconstructed using in-house code written in MATLAB (r2021a, MathWorks, Natick, Massachusetts) and templates constructed for each cohort using the mean echo image as described previously [58, 59]. Diffusion-weighted imaging (DWI) was performed using a 2D echo planar readout with three segments and parameters: TR = 4 s; TE = 25 ms; FOV = 16.32 × 10.88 mm^2^; 40 slices; resolution = 170 × 170 × 170 μm^3^; δ = 4 ms; Δ = 13 ms; 2 non-diffusion-weighted images; b-values = 2000, 6000 s/mm^2^; and 64 gradient directions. A reverse-phase-encoded image was acquired for distortion correction.

### MRI analysis

Using ITK-SNAP 4.0 software (www.itksnap.org) [60], manual segmentation of the lesion zone was completed on 3D multi-gradient echo images resized to give coronal slices 0.5 mm thick. Briefly, slice-by-slice segmentation was achieved by labelling voxels where there was an absence of tissue or abnormality compared with the contralateral hemisphere. The segmentation was subsequently used to calculate lesion volume in ITK-SNAP based on the number and size of voxels labelled. Differences in lesion size were assessed with Holm-Sidak’s multiple comparisons.

DWI processing was performed as described previously [61] using MRtrix3 [62] and FSL [63] software packages. All DWIs were registered to a study template constructed from male and female sham mice treated with vehicle. Whole-brain analyses were performed with connectivity-based fixel enhancement to investigate for differences between: *T. gondii* + CCI versus Vehicle + CCI; *T. gondii* + CCI versus *T. gondii* + Sham; and *T. gondii* + CCI versus Vehicle + Sham. Males and females were tested and presented separately and fixels with FWE corrected *p* < 0.05 considered significant.

### Neurobehavioral assessment

Beginning at 12-weeks post-injury, mice underwent a battery of behavioral tests (see Fig. 1). Except for the forced swim test, mouse behavior was objectively tracked and measured with Topscan Lite software (version 2.0; Clever Sys Inc., USA). All tests were conducted in indirect-white light during the ‘light’ cycle, and all mice were habituated to the test room for at least 15 min before beginning an assessment. All equipment was thoroughly cleaned with 80% v/v ethanol between animals.

On day 1 of testing, anxiety-like behavior was assessed in the elevated plus maze (300 mm long × 60 mm wide, closed walls 150 mm tall; maze elevated by 390 mm) as previously described [29]. Briefly, mice were placed in the center area facing an open arm and allowed to freely explore for a 5 min period. Anxiety-like behavior was quantified by the percentage of time spent in the open arms across the testing period.

On days 1 and 2 of testing, motor function was assessed with the accelerating rotarod (30 mm diameter rod, four lanes 50 mm wide each, divided by 100 mm tall partitions; Panlab/ Harvard Apparatus, Holliston, MA, USA) as previously described [29, 64]. This consisted of one day of training and one test day, with a total of 3 trials per day and an inter-trial interval of 30 min. Briefly, mice were placed on the immobile rod, before the device was switched on to rotate at 4 rotations per minute (RPM; minimum speed). Then, the rotarod accelerated over a 5 min period to a maximum speed of 40 RPM, and latency to fall from the rod was quantified.

On day 2 of testing, locomotion and anxiety-like behavior was assessed in an open field arena (400 mm long × 400 mm wide × 300 mm tall). Mice were placed in the center area of the field and allowed to freely explore across a 5 min period.

The three-chamber social test (3CT) was used to assess sociability, social exploration, social memory and social preference behaviors. As previously described [29, 64], this assessment was conducted over three consecutive 10-min stages per mouse and mice were single-housed 24 h prior to 3CT testing to promote sociability during the completion of this task. Stimulus mice used were strain, sex- and aged-matched to experimental mice, and sourced from the same in-house C57BL/6 J (Jax) colony. In the first ‘habituation’ stage, mice were placed into the arena (410 mm wide × 380 mm long × 110 mm tall) which consisted of three chambers (160 mm wide left and right chambers; 75 mm wide central chamber), with wire cages (155 mm wide × 100 mm deep × 105 mm tall) in both the left and right chamber. Mice were allowed to freely explore for 10 min before briefly returning to their home cage. In stage 2, an unfamiliar ‘stimulus 1’ mouse was then placed into one of the wire cages and the test mouse was immediately returned to the middle chamber of the arena to explore freely for 10 min. Then, in stage 3, a new unfamiliar mouse (‘stimulus 2’) was placed into the empty wire cage prior to the test mouse being returned to the arena. Again, the test mouse was allowed to freely explore all chambers for 10 min. Stages 2 and 3 assess a preference for sociability and social memory, respectively [65].

The water maze was used to assess spatial learning and memory. As previously described [29, 66], this assessment was conducted in a fiberglass tank (1200 mm diameter) filled with semi-opaque white water (22–23 °C) over 8 days and consisted of four phases: swim and escape training (visible platform; day 1), target platform location training (hidden platform; days 2–5), rest and memory consolidation (days 6–7), and probe test (day 8). All mice completed 6 trials per day for the ‘visible platform’ and ‘hidden platform’ phases, and a single trial was conducted for the probe test. Mice were given a maximum of 60 s for each trial, and the escape platform was removed for the probe trial. Number of platform location crossings (i.e., target crossings) in the probe trial was assessed as it has been shown to be a sensitive measure in the TBI setting [66, 67].

Spatial memory was also assessed using the Y-maze (380 mm long × 75 mm wide × 120 mm tall per arm) as previously described [29]. Briefly, mice were allowed to freely explore and habituate to two of the three arms for 15 min, returned to their home cage for 30 min, and then placed into the Y-maze once again where they were allowed to freely explore all arms (‘home’, ‘familiar’, and ‘novel’) for a 5 min test period. Novelty preference was calculated using the equation: novelty preference = T_novel_/T_familiar + novel_ × 100.

Last, the forced swim test was conducted to assess depression-like behavior. Mice were gently placed into a 2L beaker (~ 130 mm diameter × 123 mm water depth; 24–25 °C) and recorded with a tripod-mounted video camera for 6 min. Only the last 4 min of behavior was analyzed. The percentage of time spent immobile across this 4 min period was manually scored and used as a measure of depression-like behavior [65].

### Statistical analysis

Data was analyzed with SPSS 28.0 software (IBM Corp, Armonk, USA). Shapiro-Wilks test of normality was conducted on all data sets, and log_2_ transformations and non-parametric tests were applied when appropriate. Normally distributed data was analyzed by 2-way analysis of variance (ANOVA), with infection and injury as between-subject factors. Bonferroni post-hoc comparisons were carried out when appropriate. If data was not normally distributed it was analyzed with a Kruskal–Wallis test, with Bonferroni-corrected multiple comparisons when appropriate. Statistical significance was set as *p* < 0.05.

## Results

### Gene expression results

We conducted multiplex qPCR to examine gene expression of 49 markers (Table 1) related to pathophysiological processes involved in TBI and/or *T. gondii* infection at 2-h, 24-h, 7-days, and 18-weeks after TBI in the injured cortex. A summary of all main effects, interactions, and post-hoc comparisons related to this analysis can be found in Table 2 (2-h), Table 3 (24-h), Table 4 (7-days), and Table 5 (18-weeks) at the end of the gene expression results section, and all remaining statistical details (e.g., F, H, and p values) are in Additional file 1: Tables S1 and S2. Due to the large amount of qPCR data, the text and figures in this section will focus on statistically significant results indicating interactive/synergistic effects between a pre-existing *T. gondii* infection and TBI. However, it is important to note that in both males and females, *T. gondii* infection and CCI were found to independently alter the gene expression of other markers related to neuroinflammation, oxidative stress, neuronal integrity, BBB integrity/vascular health, and/or glutamatergic pathways in the absence of interactive effects (see Tables 2, 3, 4, 5, Additional file 1: Tables S1, S2).

**Table 2:** Results of gene expression analyses from the ipsilateral cortex at 2 h post-CCI in male and female mice

Genes	Male					Female				
	1. Vehicle + Sham (n = 5)	2.* T. gondii* + Sham (n = 5)	3. Vehicle + CCI (n = 6)	4.* T. gondii* + CCI (n = 6)	Significance	1. Vehicle + Sham (n = 6)	2.* T. gondii* + Sham (n = 6)	3. Vehicle + CCI (n = 6)	4.* T. gondii* + CCI (n = 6)	Significance
	Mean ± SD or Median [25,75 percentile]					Mean ± SD or Median [25,75 percentile]				
Neuroinflammation										
* GFAP*	0.00 ± 0.33	1.20 ± 0.57	− 0.04 ± 0.17	1.22 ± 0.54	TG	0.01 [− 0.26, 0.30]	2.12 [1.95, 2.61]	0.25 [0.12, 0.57]	2.39 [1.85, 3.01]	2 > 1, 4 > 1, 4 > 3
* CD45*	0.00 ± 0.25	2.38 ± 0.95	0.56 ± 0.25	2.53 ± 0.96	TG	− 0.17 [− 0.32, 0.31]	3.46 [3.06, 3.99]	0.34 [− 0.01, 0.66]	3.49 [2.92, 5.51]	2 > 1, 4 > 1
* CD86*	0.00 ± 0.38	1.81 ± 0.55	0.88 ± 0.24	2.12 ± 0.75	TG, CCI	0.00 ± 0.57	2.48 ± 0.40	0.78 ± 0.37	3.04 ± 0.82	TG, CCI
* TREM2*	0.00 ± 0.18	0.77 ± 0.59	− 0.22 ± 0.27	0.91 ± 0.45	TG	0.07 [− 0.20, 0.14]	1.78 [1.55, 1.91[	− 0.08 [− 0.31, 0.24]	1.78 [0.90, 2.77]	2 > 1, 4 > 1, 4 > 3, 2 > 3
* CCR5*	0.00 ± 0.61	1.42 ± 0.58	− 0.10 ± 0.28	1.59 ± 0.85	TG	0.00 ± 0.39	1.99 ± 0.33	− 0.09 ± 0.50	2.80 ± 0.94	TG
* IBA1*	0.14 [− 0.28, 0.21]	1.68 [0.70, 2.37]	− 0.08 [− 0.27, 0.09]	1.40 [0.95, 2.13]	4 > 3, 2 > 3	− 0.04 [− 0.17, 0.16]	2.80 [2.47, 2.93]	− 0.16 [− 0.52,− 0.03]	2.74 [2.26, 3.59]	4 > 3, 2 > 3
* TMEM119*	0.00 ± 0.30	0.38 ± 0.68	− 0.84 ± 0.27	0.21 ± 0.34	TG, CCI	0.00 ± 0.61	1.53 ± 0.37	− 0.31 ± 0.60	1.11 ± 0.76	TG
* GATA3*	0.00 ± 0.90	4.02 ± 1.29	1.09 ± 2.00	4.17 ± 1.03	TG	− 0.12 [− 0.42, 0.53]	5.08 [4.92, 5.34]	1.26 [0.32, 2.15]	4.94 [4.34, 6.46]	2 > 1, 4 > 1
* CXCR3*	0.00 ± 0.86	4.47 ± 1.53	− 0.35 ± 0.81	4.14 ± 1.42	TG	0.34 [− 1.11, 0.66]	6.95 [6.79, 7.41]	0.86 [− 0.60, 1.34]	6.90 [5.94, 8.24]	2 > 1, 4 > 1, 2 > 3
* STAT1*	0.14 [− 0.32, 0.25]	2.99 [1.07, 3.72]	− 0.02 [− 0.29, 0.12]	2.00 [1.50, 2.86]	2 > 1, 4 > 3, 2 > 3	− 0.05 [− 0.20, 0.19]	3.91 [3.81, 4.30]	0.04 [− 0.22, 0.66]	4.13 [3.05, 4.58]	2 > 1, 4 > 1, 4 > 3, 2 > 3
* SOCS1*	0.00 [− 0.16, 0.16]	0.94 [0.73, 2.66]	0.78 [0.09, 0.83]	1.96 [1.48, 2.52]	2 > 1, 4 > 1	0.02 [− 0.17, 0.15]	2.67 [2.29, 3.57]	0.32 [− 0.08, 0.70]	3.36 [2.40, 4.61]	2 > 1, 4 > 1, 4 > 3
* FOXP3*	0.15 [− 0.44, 0.37]	1.45 [0.59, 2.96]	− 0.24 [− 1.39, 0.14]	0.75 [0.35, 3.14]	2 > 3, 4 > 3	0.11 [− 0.66, 0.50]	3.82 [3.47, 4.63]	0.17 [− 0.13, 0.52]	4.34 [3.39, 5.92]	2 > 1, 4 > 1, 4 > 3
* TSPO*	0.05 [− 0.30, 0.27]	1.09 [0.35, 2.10]	− 0.14 [− 0.26, 0.03]	0.92 [0.64, 1.77]	2 > 3, 4 > 3	0.71 [− 0.47, 0.82]	3.17 [2.91, 3.56]	0.71 [0.39, 1.08]	3.15 [2.35, 4.28]	2 > 1, 4 > 1, 4 > 3, 2 > 3
* NLRP3*	0.00 ± 0.66	1.06 ± 0.61	3.00 ± 0.45	3.12 ± 0.86	CCI	0.00 ± 0.24	1.61 ± 0.26	2.48 ± 0.66	3.30 ± 0.63	TG, CCI
* IL1β*	0.00 ± 2.25	2.61 ± 1.42	4.32 ± 0.39	4.71 ± 1.36	TG, CCI	0.00 ± 1.59	2.36 ± 1.16	3.40 ± 0.74	5.15 ± 0.89	TG, CCI
* IL2*	− 0.03 [− 0.37, 0.38]	2.02 [0.14, 3.95]	0.11 [− 0.29, 0.30]	2.76 [1.45, 4.24]	ns	− 0.46 [− 0.77, 0.73]	3.92 [3.62, 4.69]	− 0.33 [− 1.29, 0.09]	4.50 [4.09, 7.12]	4 > 1, 4 > 3
* IL6*	− 1.07 [− 1.38, 1.91]	2.38 [0.87, 2.62]	2.75 [2.54, 3.28]	3.69 [1.82, 4.15]	4 > 1	0.00 ± 1.11	1.35 ± 0.84	2.58 ± 0.76	3.94 ± 0.74	TG, CCI
* IL10*	− 0.57 [− 0.65, 0.94]	3.49 [2.73, 4.95]	3.46 [2.83, 4.27]	4.55 [3.88, 5.59]	4 > 1	0.00 ± 1.42	4.02 ± 0.97	2.46 ± 0.75	5.46 ± 1.12	TG, CCI
* IL12p40*	− 1.71 [− 2.36, 3.22]	4.16 [3.59, 5.38]	6.63 [5.66, 7.03]	7.36 [6.61, 7.71]	3 > 1, 4 > 1	0.42 [− 0.78, 1.03]	3.44 [2.92, 4.08]	3.59 [2.79, 3.82]	5.27 [4.83, 5.63]	4 > 1
* IL33*	0.00 ± 0.38	0.32 ± 0.57	− 0.05 ± 0.36	0.32 ± 0.65	ns	0.00 ± 0.42	0.75 ± 0.58	− 0.16 ± 0.36	0.18 ± 0.55	TG
* TNFα*	− 1.43 [− 1.80, 2.51]	3.21 [2.05, 3.52]	5.82 [5.35, 6.12]	5.76 [4.62, 6.30]	3 > 1, 4 > 1	0.66 [− 1.04, 1.39]	2.73 [2.01, 3.00]	4.09 [3.49, 4.49]	4.48 [3.93, 5.26]	3 > 1, 4 > 1
* CSF1*	0.00 ± 0.26	0.41 ± 0.70	0.33 ± 0.29	0.77 ± 0.48	TG	0.00 ± 0.25	1.40 ± 0.35	0.62 ± 0.46	1.71 ± 0.44	TG, CCI
* CSF2*	0.00 ± 1.38	2.39 ± 1.48	2.72 ± 0.55	3.60 ± 1.42	TG, CCI	0.00 ± 1.01	3.36 ± 0.45	1.81 ± 0.92	4.73 ± 1.21	TG, CCI
* CXCL10*	0.00 ± 2.06	5.73 ± 1.69	3.45 ± 0.31	6.41 ± 1.36	TG, CCI, *	0.00 ± 0.97	6.83 ± 1.21	2.92 ± 0.73	7.92 ± 1.06	TG, CCI, *
* IFNγ*	− 0.84 [− 0.92, 1.34]	6.53 [5.69, 7.99]	− 0.08 [− 0.51, 1.12]	7.97 [6.48, 9.15]	4 > 1, 4 > 3	− 0.36 [− 0.92, 1.12]	8.93 [8.26,10.26]	0.88 [− 0.31, 2.01]	10.52 [9.47, 12.31]	2 > 1, 4 > 1, 4 > 3
* TGFβ*	0.00 ± 0.79	1.58 ± 0.57	1.61 ± 0.26	2.23 ± 0.83	TG, CCI	0.00 ± 0.32	1.83 ± 0.19	1.09 ± 0.47	2.89 ± 0.89	TG, CCI
* ARG1*	0.27 [− 0.93, 0.80]	0.38 [− 1.04, 3.24]	0.08 [− 0.62, 0.62]	0.96 [− 0.40, 3.13]	ns	0.04 [− 0.71, 0.72]	2.11 [1.11, 3.05]	0.57 [0.15, 1.16]	2.79 [1.55, 7.40]	4 > 1
* CCL2*	− 0.56 [− 1.65, 1.93]	2.63 [1.78, 3.41]	3.26 [2.90, 3.63]	3.46 [2.59, 4.22]	3 > 1, 4 > 1	0.58 [− 0.75, 1.12]	2.09 [1.14, 2.72]	2.28 [1.68, 2.68]	3.61 [2.77, 4.95]	4 > 1
* CCL4*	− 1.56 [− 2.69, 3.47]	3.78 [1.28, 4.14]	5.62 [4.74, 5.90]	5.72 [4.79, 6.31]	3 > 1, 4 > 1	0.39 [− 0.69, 1.15]	1.13 [0.45, 2.05]	4.55 [4.08, 4.93]	4.94 [4.43, 5.53]	3 > 1, 4 > 1, 4 > 2
* CCL5*	0.00 ± 0.74	5.75 ± 1.75	1.87 ± 0.40	6.18 ± 1.45	TG, CCI	0.19 [− 0.27, 0.27]	7.13 [6.95, 8.09]	1.76 [0.94, 3.29]	7.71 [7.43, 10.15]	2 > 1, 4 > 1
* CCL12*	0.00 ± 0.85	3.24 ± 1.33	2.39 ± 0.53	4.21 ± 0.64	TG, CCI	0.00 ± 0.60	3.91 ± 0.61	2.00 ± 0.46	4.65 ± 0.87	TG, CCI, *
Neuronal cell markers										
* MAP2*	0.00 ± 0.16	− 0.11 ± 0.23	− 0.09 ± 0.18	− 0.03 ± 0.18	ns	0.00 ± 0.32	− 0.37 ± 0.16	− 0.12 ± 0.16	− 0.40 ± 0.14	TG
Oxidative stress										
* IDO1*	− 0.12 [− 0.20, 0.26]	6.61 [6.55, 7.64]	0.60 [0.22, 1.83]	6.00 [5.37, 7.42]	2 > 1, 4 > 1	− 0.46 [− 0.77, 0.73]	8.46 [7.71, 8.90]	− 0.33 [− 1.29, 0.64]	8.16 [6.96, 9.72]	2 > 1, 4 > 1, 4 > 3, 2 > 3
* NOS2*	− 0.13 [− 0.27, 0.33]	1.46 [0.00, 4.05]	− 0.07 [− 0.33, 0.14]	0.92 [− 0.05, 2.84]	ns	− 0.04 [− 0.22, 0.19]	3.74 [2.65, 5.66]	0.04 [− 0.21, 0.19]	4.68 [3.24, 7.24]	2 > 1, 4 > 1, 4 > 3, 2 > 3
* CYBB*	0.00 ± 0.67	3.56 ± 1.50	0.61 ± 0.36	3.43 ± 1.47	TG	0.13 [− 0.46, 0.30]	5.36 [4.99, 5.56]	0.75 [− 0.03, 1.12]	5.08 [4.70, 6.97]	2 > 1, 4 > 1
Glutamate pathway										
* PHGDH*	0.00 ± 0.19	− 0.05 ± 0.30	− 0.14 ± 0.22	0.13 ± 0.30	ns	0.00 ± 0.39	0.16 ± 0.26	0.16 ± 0.28	0.03 ± 0.21	ns
* GLT1*	0.00 ± 0.10	− 0.29 ± 0.31	− 0.11 ± 0.21	− 0.22 ± 0.26	ns	0.00 ± 0.51	− 0.21 ± 0.23	0.06 ± 0.20	− 0.49 ± 0.41	TG
* GLUL*	0.00 ± 0.13	− 0.05 ± 0.28	− 0.09 ± 0.31	0.03 ± 0.31	ns	0.00 ± 0.19	− 0.16 ± 0.25	− 0.06 ± 0.29	− 0.49 ± 0.23	TG, CCI
* GRIN2B*	0.00 ± 0.29	− .010 ± 0.34	− 0.08 ± 0.37	− 0.19 ± 0.40	ns	0.00 ± 0.40	− 0.36 ± 0.34	− 0.36 ± 0.36	− 0.73 ± 0.37	TG, CCI
* GAD1*	0.00 ± 0.36	− 0.14 ± 0.42	− 0.16 ± 0.27	− 0.19 ± 0.38	ns	0.00 ± 0.18	− 0.02 ± 0.43	− 0.09 ± 0.56	− 0.39 ± 0.43	ns
* GLAST*	0.00 ± 0.18	− 0.03 ± 0.39	− 0.13 ± 0.23	0.19 ± 0.13	ns	0.00 ± 0.23	0.40 ± 0.17	− 0.04 ± 0.29	0.35 ± 0.35	TG
* PPARγ*	0.00 ± 0.31	0.05 ± 0.41	0.09 ± 0.22	− 0.16 ± 0.79	ns	0.00 ± 0.45	0.14 ± 0.39	− 0.20 ± 0.55	− 0.67 ± 0.55	CCI

**Fig. 3:**
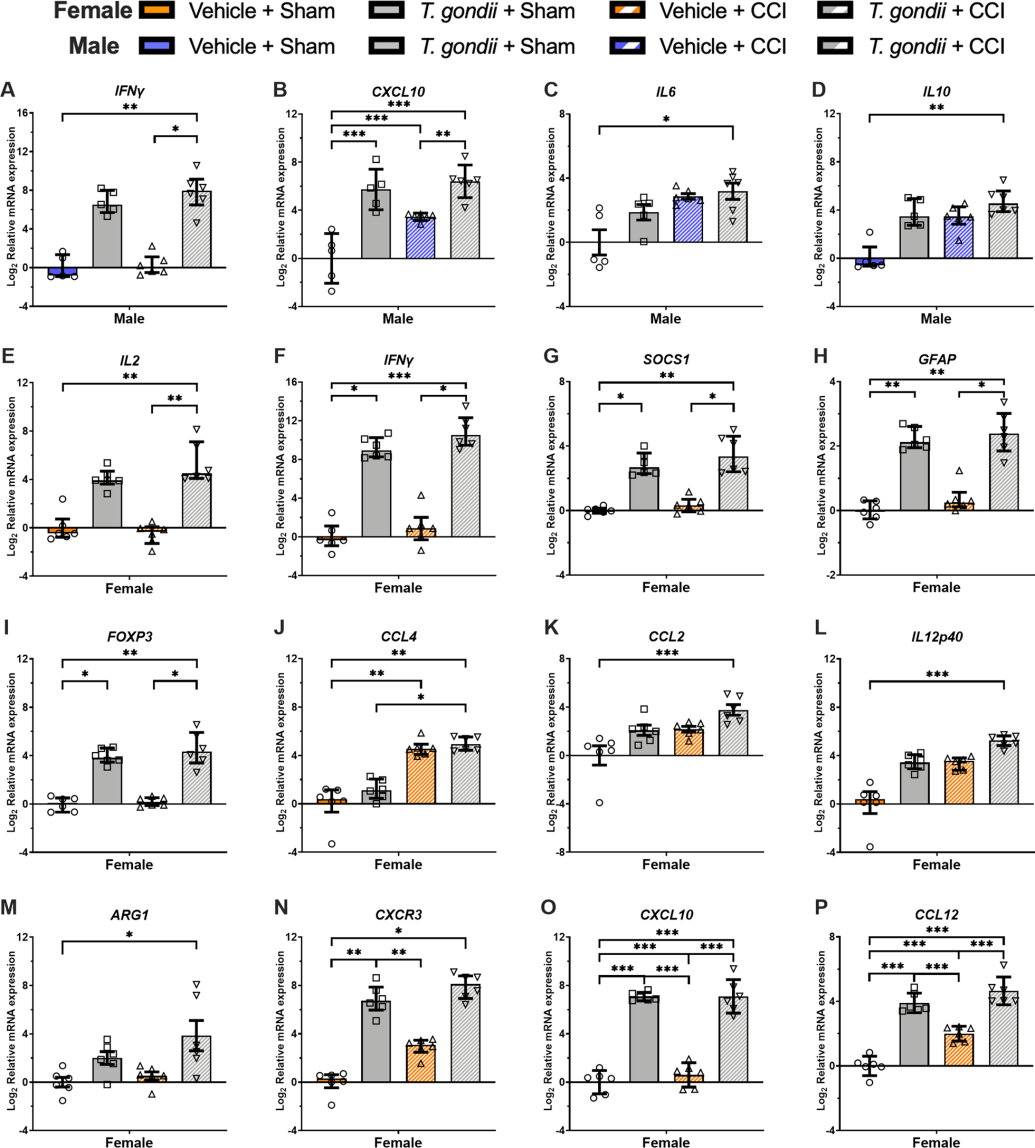
Gene expression findings from the ipsilateral cortex suggesting interactive effects of *T.gondii* and CCI at 2-h post-injury in male and female mice. In both males **(A–D)** and females **(E–P)**, there was evidence that combined *T. Gondii* and CCI resulted in altered pathophysiology relative to *T. gondii* and/or CCI in isolation at 2-h post-injury. This includes genes reflective of inflammatory mediators (**A–G, I–P)**, and astrogliosis **(H)**. **B, O** and **P** were analyzed with 2-way ANOVA and the significant *T. Gondii* * CCI interaction was further analyzed with Bonferroni post-hoc comparisons. These normally distributed data are displayed as mean ± SD. Data in **A** and **C–N** were not normally distributed and were therefore analyzed with Kruskal–Wallis tests and presented as median with interquartile range. *p < 0.05, **p ≤ 0.01. ***p ≤ 0.001

Normally distributed data were analyzed with a 2-way ANOVA and are presented as mean ± SD. Under the ‘Significance’ column, ‘TG’ indicates a significant ANOVA main effect for *T. gondii*, ‘CCI’ indicates a significant ANOVA main effect for CCI, and ‘*’ indicates a significant ANOVA interaction between *T. gondii* and CCI. Post-hoc findings of interactive effects are detailed in the results section and Fig. 3. Non-normally distributed data were analyzed with Kruskal–Wallis tests and are presented as median [25,75 percentile]. Under the ‘Significance’ column, group differences identified with Bonferroni-corrected comparisons are summarized using the following group labels: 1 = Vehicle + Sham, 2 = *T. gondii* + Sham, 3 = Vehicle + CCI, 4 = *T. gondii* + CCI. ns = not statistically significant (i.e., *p* > .05)

**Table 3:** Results of gene expression analyses from the ipsilateral cortex at 24 h post-CCI in male and female mice

Genes	Male					Female				
	1. Vehicle + Sham (n = 5)	2.* T. gondii* + Sham (n = 6)	3. Vehicle + CCI (n = 6)	4.* T. gondii* + CCI (n = 6)	Significance	1. Vehicle + Sham (n = 5)	2.* T. gondii* + Sham (n = 6)	3. Vehicle + CCI (n = 6)	4.* T. gondii* + CCI (n = 6)	Significance
	Mean ± SD or Median [25,75 percentile]					Mean ± SD or Median [25,75 percentile]				
Neuroinflammation										
* GFAP*	0.00 ± 0.39	0.17 ± 0.53	1.00 ± 0.42	0.91 ± 0.17	CCI	− 0.03 [− 0.68, 0.70]	0.87 [0.65, 1.64]	1.94 [1.78, 2.37]	2.09 [1.89, 2.18]	3 > 1, 4 > 1
* CD45*	0.00 ± 0.19	3.00 ± 0.77	1.08 ± 0.33	3.41 ± 0.91	TG, CCI	0.00 ± 0.30	2.27 ± 0.92	1.56 ± 0.96	3.59 ± 0.57	TG, CCI
* CD86*	0.00 [− 0.11, 0.11]	2.51 [2.21, 2.94]	0.50 [0.42, 0.62]	3.26 [2.49, 3.60]	2 > 1, 4 > 1	0.12 [− 0.27, 0.20]	2.08 [0.93, 2.71]	0.59 [0.37, 1.15]	3.22 [2.59, 3.38]	2 > 1, 4 > 1
* CD206*	− 0.24 [− 0.67, 0.79]	1.11 [0.98, 1.33]	0.86 [0.39, 1.50]	0.88 [0.64, 1.37]	ns	0.00 ± 0.51	0.63 ± 0.34	0.28 ± 0.44	0.67 ± 0.50	TG
* TREM2*	0.00 ± 0.22	1.39 ± 0.64	0.43 ± 0.27	1.72 ± 0.72	TG	0.00 ± 0.12	1.00 ± 0.61	0.19 ± 0.42	1.65 ± 0.38	TG, CCI
* CCR5*	0.00 ± 0.25	1.15 ± 0.52	0.56 ± 0.28	1.75 ± 0.73	TG, CCI	0.00 ± 0.43	1.23 ± 0.70	1.06 ± 0.35	2.36 ± 0.29	TG, CCI
* IBA1*	0.00 ± 0.14	2.40 ± 0.67	0.66 ± 0.24	2.79 ± 0.66	TG, CCI	0.10 [− 0.18, 0.13]	1.83 [0.76, 2.42]	0.76 [0.57, 0.85]	2.67 [2.49, 3.07]	2 > 1, 4 > 1
* TMEM119*	0.00 ± 0.36	0.84 ± 0.68	− 0.02 ± 0.18	0.89 ± 0.52	TG	0.03 [− 0.17, 0.15]	0.52 [0.06, 1.30]	− 0.08 [− 0.62, 0.01]	0.93 [0.59, 1.15]	4 > 3
* GATA3*	− 0.04 [− 1.34, 1.36]	5.88 [5.17, 6.84]	0.95 [− 0.02, 1.96]	6.25 [5.46, 6.73]	2 > 1, 4 > 1, 4 > 3	0.00 ± 0.78	4.30 ± 0.96	1.01 ± 0.93	5.20 ± 0.86	TG, CCI
* CXCR3*	0.20 [− 1.12, 1.02]	6.18 [5.67, 7.17]	0.68 [0.32, 1.13]	6.51 [5.79, 6.95]	2 > 1, 4 > 1, 4 > 3, 2 > 3	0.09 [− 0.21, 0.16]	4.62 [4.04, 6.28]	− 0.12 [− 0.70, 0.88]	5.65 [5.41, 7.14]	4 > 1, 4 > 3
* STAT1*	0.01 [− 0.17, 0.16]	3.46 [3.12, 4.26]	0.45 [0.37, 0.78]	3.81 [3.26, 4.29]	2 > 1, 4 > 1	0.09 [− 0.28, 0.23]	2.79 [1.59, 3.62]	0.37 [0.28, 0.54]	3.23 [2.96, 3.67]	2 > 1, 4 > 1
* SOCS1*	0.00 ± 0.53	2.14 ± 0.87	0.16 ± 0.30	2.92 ± 1.24	TG	− 0.16 [− 0.41, 0.49]	1.08 [0.20, 2.88]	0.18 [0.14, 0.41]	2.38 [2.04, 2.98]	4 > 1
* FOXP3*	− 0.32 [− 0.41, 0.57]	1.62 [0.77, 3.07]	− 0.04 [− 0.61, 0.37]	2.68 [2.04, 3.61]	4 > 1, 4 > 3	0.00 ± 1.11	1.96 ± 1.45	0.76 ± 0.79	3.27 ± 0.91	TG, CCI
* TSPO*	0.00 ± 0.38	1.18 ± 1.35	0.73 ± 0.23	2.61 ± 0.77	TG, CCI	0.08 [− 0.17, 0.13]	0.93 [0.09, 2.04]	0.57 [− 0.76, 0.80]	2.49 [2.24, 2.79]	4 > 1, 4 > 3
* NLRP3*	0.00 ± 0.30	1.65 ± 0.50	0.85 ± 0.30	2.10 ± 0.67	TG, CCI	0.00 ± 0.48	1.33 ± 0.84	1.12 ± 0.47	2.09 ± 0.27	TG, CCI
* IL1β*	0.00 ± 0.91	2.06 ± 1.51	1.16 ± 0.70	3.02 ± 1.39	TG, CCI	0.00 ± 1.22	1.63 ± 1.80	2.23 ± 0.91	3.43 ± 0.82	TG, CCI
* IL2*	− 0.22 [− 0.48, 0.59]	3.32 [2.36, 4.61]	0.46 [0.11, 0.67]	4.02 [2.70, 4.88]	2 > 1, 4 > 1, 4 > 3	0.13 [-0.49, 0.43]	2.60 [1.93, 3.91]	0.09 [− 0.33, 1.76]	2.66 [1.71, 4.12]	ns
* IL6*	0.00 ± 0.89	0.56 ± 1.47	2.12 ± 0.81	2.72 ± 1.00	CCI	0.00 ± 0.96	1.06 ± 1.71	3.37 ± 1.74	4.02 ± 0.94	CCI
* IL10*	0.00 ± 0.56	4.94 ± 0.93	1.06 ± 1.02	5.59 ± 1.26	TG	− 0.24 [− 0.89, 1.01]	3.32 [0.88, 3.79]	0.29 [− 0.54, 1.96]	4.94 [4.58, 5.29]	4 > 1, 4 > 3
* IL12p40*	0.61 [− 1.13, 0.83]	6.17 [5.74, 7.64]	1.39 [− 0.15, 1.59]	7.27 [5.55, 8.33]	2 > 1, 4 > 1, 4 > 3	0.00 ± 0.97	4.24 ± 1.98	0.49 ± 1.88	5.83 ± 0.94	TG
* IL33*	0.14 [− 0.41, 0.34]	− 0.15 [− 0.37, 0.23]	0.33 [− 0.17, 0.98]	0.32 [0.10, 0.67]	ns	0.00 ± 0.24	0.60 ± 0.41	1.21 ± 0.33	1.37 ± 0.24	TG, CCI
* TNFα*	0.00 ± 0.54	2.38 ± 0.85	1.29 ± 0.49	3.73 ± 1.15	TG, CCI	0.00 ± 1.45	2.92 ± 1.59	3.10 ± 0.40	4.94 ± 0.59	TG, CCI
* CSF1*	0.00 ± 0.21	0.81 ± 0.68	0.63 ± 0.36	1.21 ± 0.47	TG, CCI	0.00 ± 0.25	0.61 ± 0.49	0.69 ± 0.59	1.18 ± 0.22	TG, CCI
* CSF2*	0.00 ± 1.12	4.26 ± 1.10	1.71 ± 1.38	4.53 ± 1.05	TG	0.00 ± 0.95	2.15 ± 1.15	0.72 ± 1.57	3.23 ± 0.82	TG
* CXCL10*	0.00 ± 0.87	4.57 ± 1.16	1.12 ± 0.91	6.09 ± 0.85	TG, CCI	0.00 ± 1.56	4.66 ± 2.13	2.17 ± 0.24	6.54 ± 0.33	TG, CCI
* IFNγ*	− 0.05 [− 0.32, 0.34]	8.44 [7.57, 10.08]	0.77 [0.58, 0.92]	8.97 [7.04, 10.68]	2 > 1, 4 > 1	− 0.21 [− 0.31, 0.42]	6.70 [5.32, 8.88]	0.05 [− 0.14, 1.95]	7.50 [7.23, 8.66]	2 > 1, 4 > 1, 4 > 3
* TGFβ*	− 0.12 [− 0.66, 0.72]	0.86 [0.36, 1.75]	0.55 [0.22, 0.97]	1.80 [1.06, 2.41]	4 > 1	0.00 ± 0.90	1.03 ± 0.70	1.64 ± 0.95	2.87 ± 0.46	TG, CCI
* ARG1*	0.00 ± 0.78	− 1.73 ± 1.37	3.05 ± 0.98	3.15 ± 1.43	CCI	0.00 [− 2.01, 2.01]	− 0.29 [− 1.31, 0.26]	4.81 [4.52, 5.51]	5.98 [4.57, 6.84]	4 > 1, 3 > 2, 4 > 2
* CCL2*	0.00 ± 0.34	1.51 ± 1.36	1.77 ± 0.60	3.91 ± 0.92	TG, CCI	0.00 ± 1.45	2.79 ± 2.26	3.66 ± 0.58	5.62 ± 0.61	TG, CCI
* CCL4*	0.00 ± 0.81	1.42 ± 1.18	2.66 ± 0.80	3.26 ± 0.75	TG, CCI	− 0.62 [− 1.27, 1.58]	1.61 [0.48, 3.28]	4.54 [4.22, 5.02]	5.03 [4.34, 5.16]	3 > 1, 4 > 1
* CCL5*	0.22 [− 0.48, 0.37]	6.70 [6.07, 7.75]	1.29 [0.92, 1.85]	6.92 [6.06, 8.58]	2 > 1, 4 > 1	0.13 [− 0.37, 0.31]	5.30 [3.55, 6.87]	0.86 [0.36, 1.64]	6.67 [5.98, 7.97]	2 > 1, 4 > 1, 4 > 3
* CCL12*	− 0.12 [− 0.33, 0.39]	1.05 [− 0.03, 1.70]	0.56 [0.24, 1.02]	1.80 [1.24, 3.14]	4 > 1	0.00 ± 1.62	2.30 ± 1.64	2.59 ± 0.95	3.89 ± 0.50	TG, CCI
Neuronal cell markers										
* MAP2*	0.00 [− 0.20, 0.19]	0.19 [0.08, 0.30]	0.18 [0.06, 0.24]	− 0.18 [− 0.53, − 0.09]	2 > 4	− 0.04 [− 0.14, 0.16]	0.07 [− 0.08, 0.11]	− 0.01 [− 0.26, 0.30]	− 0.36 [− 0.61, − 0.23]	2 > 4
Oxidative stress										
* IDO1*	− 0.16 [− 0.42, 0.50]	6.59 [5.62, 7.56]	0.52 [0.17, 0.73]	7.50 [5.54, 8.78]	2 > 1, 4 > 1, 4 > 3	0.13 [− 0.49, 0.43]	5.47 [3.99, 6.67]	− 0.14 [− 0.33, 1.76]	7.68 [7.04, 8.53]	4 > 1, 4 > 3
* NOS2*	0.03 [− 0.39, 0.38]	2.80 [1.94, 4.78]	0.59 [0.48, 0.94]	4.43 [2.03, 5.60]	2 > 1, 4 > 1	− 0.09 [− 0.31, 0.35]	1.17 [0.01, 3.87]	0.68 [0.33, 1.24]	2.66 [2.01, 3.41]	4 > 1
* CYBB*	0.15 [− 0.37, 0.30]	5.22 [4.50, 6.01]	1.13 [1.07, 1.24]	5.66 [4.47, 6.32]	2 > 1, 4 > 1	0.19 [− 0.46, 0.36]	4.04 [2.70, 5.24]	0.96 [0.90, 1.64]	5.43 [4.85, 6.39]	2 > 1, 4 > 1, 4 > 3
Glutamate pathway										
* PHGDH*	0.00 ± 0.49	0.41 ± 0.61	0.40 ± 0.31	0.33 ± 0.31	ns	0.00 ± 0.51	0.08 ± 0.14	− 0.07 ± 0.27	− 0.39 ± 0.17	CCI
* GLT1*	0.00 ± 0.36	0.17 ± 0.45	0.04 ± 0.23	− 0.38 ± 0.34	ns	0.12 [− 0.34, 0.28]	− 0.08 [− 0.11, 0.02]	− 0.36 [− 0.86, − 0.13]	− 0.91 [− 1.17, − 0.66]	1 > 4, 2 > 4
* GLUL*	0.00 ± 0.58	1.00 ± 0.30	0.27 ± 0.38	0.23 ± 0.34	TG, *	0.00 ± 0.62	0.14 ± 0.27	− 0.49 ± 0.50	− 0.78 ± 0.31	CCI
* GRIN2B*	0.00 ± 0.20	0.36 ± 0.25	0.32 ± 0.32	− 0.08 ± 0.34	*	− 0.01 [− 0.38, 0.39]	0.04 [− 0.03, 0.26]	− 0.06 [− 0.24, 0.07]	− 0.41 [− 0.82, − 0.12]	ns
* GAD1*	0.00 ± 0.22	− 0.42 ± 0.55	− 0.34 ± 0.29	− 0.78 ± 0.39	TG	0.05 [− 0.24, 0.21]	− 0.18 [− 0.31, 0.02]	− 0.30 [− 0.45, 0.09]	− 0.55 [− 1.12, − 0.19]	ns
* GLAST*	0.00 ± 0.17	0.75 ± 0.61	0.29 ± 0.22	0.63 ± 0.26	TG	0.00 ± 0.24	0.26 ± 0.20	− 0.10 ± 0.31	0.26 ± 0.10	TG
* PPARγ*	0.00 ± 0.30	− 0.15 ± 0.17	− 0.41 ± 0.27	− 0.45 ± 0.35	CCI	0.00 ± 0.15	− 0.27 ± 0.23	− 0.38 ± 0.17	− 0.43 ± 0.40	CCI

Normally distributed data were analyzed with a 2-way ANOVA and are presented as mean ± SD. Under the ‘Significance’ column, ‘TG’ indicates a significant ANOVA main effect for *T. gondii*, ‘CCI’ indicates a significant ANOVA main effect for CCI, and ‘*’ indicates a significant ANOVA interaction between *T. gondii* and CCI. Post-hoc findings of interactive effects are detailed in the results section and Fig. 3. Non-normally distributed data were analyzed with Kruskal–Wallis tests and are presented as median [25,75 percentile]. Under the ‘Significance’ column, group differences identified with Bonferroni-corrected comparisons are summarized using the following group labels: 1 = Vehicle + Sham, 2 = *T. gondii* + Sham, 3 = Vehicle + CCI, 4 = *T. gondii* + CCI. ns = not statistically significant (i.e., *p* > .05)

**Table 4:** Results of gene expression analyses from the ipsilateral cortex at 7 days post-CCI in male and female mice

Genes	Male					Female				
	1. Vehicle + Sham (n = 6)	2. *T. gondii* + Sham (n = 6)	3. Vehicle + CCI (n = 6)	4. *T. gondii* + CCI (n = 6)	Significance	1. Vehicle + Sham (n = 6)	2. *T. gondii* + Sham (n = 6)	3. Vehicle + CCI (n = 6)	4. *T. gondii* + CCI (n = 6)	Significance
	Mean ± SD or Median [25,75 percentile]					Mean ± SD or Median [25,75 percentile]				
Neuroinflammation										
* GFAP*	0.00 ± 0.91	0.46 ± 0.75	2.07 ± 0.25	1.94 ± 0.49	CCI	0.00 ± 1.46	0.78 ± 0.53	2.33 ± 0.71	2.31 ± 0.67	CCI
* CD45*	0.00 ± 0.40	1.67 ± 1.63	1.38 ± 0.26	3.23 ± 1.07	TG, CCI	0.00 ± 0.80	2.33 ± 1.31	1.55 ± 0.45	2.96 ± 0.91	TG, CCI
* CD86*	0.00 ± 0.35	1.29 ± 1.21	1.11 ± 0.37	2.62 ± 0.84	TG, CCI	0.00 ± 0.60	1.58 ± 1.00	1.19 ± 0.34	2.39 ± 0.78	TG, CCI
* CD206*	0.00 ± 0.40	0.10 ± 0.33	0.35 ± 0.32	0.47 ± 0.56	CCI	0.00 ± 0.57	0.28 ± 0.43	0.37 ± 0.34	0.82 ± 0.56	CCI
* TREM2*	0.00 ± 0.41	0.48 ± 0.57	1.62 ± 0.29	1.98 ± 0.40	TG, CCI	0.00 ± 0.67	0.96 ± 0.46	1.81 ± 0.39	2.20 ± 0.86	TG, CCI
* CCR5*	0.00 ± 0.28	0.88 ± 1.07	0.88 ± 0.28	1.78 ± 0.73	TG, CCI	0.00 ± 0.49	1.13 ± 0.87	1.01 ± 0.25	1.81 ± 0.72	TG, CCI
* IBA1*	0.00 ± 0.29	1.42 ± 1.12	1.28 ± 0.33	2.64 ± 0.82	TG, CCI	0.00 ± 0.78	1.62 ± 0.92	1.52 ± 0.35	2.74 ± 0.79	TG, CCI
* TMEM119*	0.00 ± 0.24	0.71 ± 0.53	0.70 ± 0.32	1.47 ± 0.43	TG, CCI	0.00 ± 0.66	1.29 ± 0.33	0.99 ± 0.23	1.69 ± 0.35	TG, CCI
* GATA3*	0.00 ± 0.83	2.58 ± 1.91	0.20 ± 1.72	3.95 ± 1.17	TG	0.00 ± 1.58	3.32 ± 1.40	0.43 ± 0.83	3.79 ± 0.77	TG
* CXCR3*	0.02 [− 0.36, 0.34]	4.17 [2.41, 4.89]	1.11 [0.90, 1.69]	5.49 [4.71, 6.40]	2 > 1, 4 > 1	− 0.25 [− 0.51, 0.39]	4.46 [3.65, 6.00]	0.99 [0.59, 1.25	5.02 [4.18, 5.50]	2 > 1, 4 > 1
* STAT1*	0.00 ± 0.31	2.23 ± 1.58	1.08 ± 0.40	3.53 ± 1.39	TG, CCI	0.00 ± 0.70	2.31 ± 1.30	0.86 ± 0.35	3.28 ± 0.96	TG, CCI
* SOCS1*	0.10 [− 0.25, 0.27]	1.01 [0.57, 2.67]	0.47 [-0.04, 0.76]	2.79 [1.30, 4.21]	4 > 1	− 0.01 [− 0.31, 0.22]	1.65 [0.52, 3.48]	0.35 [0.11, 0.72]	1.72 [1.27, 2.45]	2 > 1, 4 > 1
* FOXP3*	0.04 [− 0.56, 0.47]	1.79 [1.41, 2.78]	0.78 [-0.05, 1.19]	4.11 [2.44, 5.40]	4 > 1, 4 > 3	0.00 ± 0.93	3.56 ± 1.67	0.68 ± 0.76	3.44 ± 1.09	TG
* TSPO*	0.00 ± 0.60	0.61 ± 1.25	1.05 ± 0.58	2.49 ± 1.11	TG, CCI	0.00 ± 1.04	1.85 ± 0.97	1.40 ± 0.46	2.44 ± 0.83	TG, CCI
* NLRP3*	0.00 ± 0.33	0.94 ± 1.10	1.05 ± 0.13	2.19 ± 0.64	TG, CCI	0.00 ± 0.52	1.61 ± 1.11	1.19 ± 0.41	2.16 ± 0.63	TG, CCI
* IL1β*	0.00 ± 0.90	2.10 ± 2.25	1.23 ± 0.91	2.99 ± 1.80	TG	0.00 ± 1.14	3.50 ± 2.97	2.10 ± 0.87	2.65 ± 1.24	TG
* IL2*	− 0.08 [− 0.32, 0.47]	1.02 [− 0.02, 2.99]	− 0.83 [− 1.09, 0.16]	3.49 [2.14, 4.77]	4 > 3	− 0.05 [− 0.57, 0.58]	3.69 [0.06, 4.92]	− 0.67 [− 0.86, − 0.22]	2.10 [0.72, 2.94]	2 > 3
* IL6*	0.00 ± 0.54	1.27 ± 1.26	0.84 ± 0.97	1.83 ± 1.27	TG	0.03 [− 0.42, 0.42]	1.58 [0.51, 3.47]	0.44 [0.18, 1.07]	0.81 [0.64, 1.20]	2 > 1
* IL10*	0.00 ± 0.47	2.79 ± 2.06	0.16 ± 1.79	3.96 ± 1.49	TG	0.00 ± 0.44	3.38 ± 1.56	0.76 ± 3.19	3.58 ± 1.07	TG
* IL12p40*	0.00 ± 1.29	3.11 ± 2.31	0.68 ± 1.04	4.59 ± 1.42	TG	0.00 ± 1.48	4.70 ± 2.50	0.57 ± 0.54	3.48 ± 1.04	TG
* IL33*	0.00 ± 0.38	-0.11 ± 0.33	0.20 ± 0.38	0.41 ± 0.40	CCI	0.00 ± 0.25	0.18 ± 0.33	0.45 ± 0.40	0.53 ± 0.64	CCI
* TNFα*	0.00 ± 1.07	2.62 ± 2.58	1.66 ± 0.34	4.53 ± 1.60	TG, CCI	0.00 ± 1.58	4.19 ± 2.06	2.58 ± 1.08	4.30 ± 1.28	TG, CCI
* CSF1*	0.00 ± 0.29	0.16 ± 0.53	0.60 ± 0.15	1.23 ± 0.63	TG, CCI	0.00 ± 0.45	0.85 ± 0.43	0.95 ± 0.34	1.41 ± 0.63	TG, CCI
* CSF2*	0.00 ± 0.62	1.82 ± 2.04	1.00 ± 1.69	3.15 ± 1.14	TG	0.00 ± 0.62	3.84 ± 2.32	1.76 ± 0.79	3.40 ± 0.79	TG
* CXCL10*	0.00 ± 1.19	2.44 ± 2.40	2.37 ± 0.70	4.71 ± 1.79	TG, CCI	0.00 ± 1.77	5.03 ± 2.37	3.03 ± 0.56	5.32 ± 1.31	TG, CCI
* IFNγ*	0.12 [− 0.37, 0.53]	5.53 [4.11, 6.81]	− 0.13 [− 0.59, 0.46]	7.69 [6.06, 9.24]	4 > 1, 4 > 3	− 0.22 [− 0.92, 1.10]	6.52 [4.20, 8.68]	− 0.39 [− 0.83, 0.10]	5.80 [4.57, 7.00]	2 > 1, 4 > 1, 2 > 3, 4 > 3
* TGFβ*	0.00 ± 0.39	0.82 ± 1.02	1.12 ± 0.26	2.18 ± 0.77	TG, CCI	0.00 ± 0.49	1.21 ± 0.92	1.34 ± 0.41	2.18 ± 0.97	TG, CCI
* ARG1*	0.00 ± 1.19	1.53 ± 3.37	2.65 ± 1.43	3.04 ± 2.52	CCI	0.00 ± 0.92	0.88 ± 1.90	2.05 ± 0.81	2.59 ± 2.24	CCI
* CCL2*	0.00 ± 0.95	1.90 ± 2.12	1.56 ± 0.69	3.94 ± 1.68	TG, CCI	0.00 ± 1.57	4.04 ± 2.65	2.51 ± 0.63	4.60 ± 1.29	TG, CCI
* CCL4*	0.00 ± 1.23	0.47 ± 1.37	1.96 ± 0.15	2.24 ± 0.99	CCI	0.00 ± 1.85	2.49 ± 1.96	3.18 ± 0.65	2.91 ± 1.06	CCI, *
* CCL5*	0.00 ± 1.18	3.53 ± 2.33	1.70 ± 0.47	5.59 ± 1.75	TG, CCI	0.00 ± 1.99	5.21 ± 2.13	2.20 ± 0.38	5.32 ± 1.50	TG
* CCL12*	0.00 ± 0.75	1.51 ± 1.59	1.45 ± 0.76	3.01 ± 0.91	TG, CCI	0.00 ± 1.15	2.91 ± 1.53	2.16 ± 0.59	3.70 ± 0.95	TG, CCI
Neuronal cell markers										
* MAP2*	0.00 ± 0.18	− 0.06 ± 0.19	− 0.14 ± 0.16	− 0.28 ± 0.29	ns	0.00 ± 0.15	− 0.19 ± 0.23	− 0.21 ± 0.21	− 0.34 ± 0.24	ns
Oxidative stress										
* IDO1*	− 0.13 [− 0.67, 0.72]	3.27 [1.81, 4.84]	− 0.41 [− 0.92, 0.18]	5.72 [4.97, 7.45]	4 > 1, 4 > 3	− 0.18 [− 0.50, 0.65]	5.16 [3.95, 6.55]	− 0.61 [− 0.80, − 0.15]	4.49 [3.58, 5.53]	2 > 1, 2 > 3, 4 > 3
* NOS2*	0.30 [− 0.15, 0.20]	2.00 [0.38, 2.92]	0.25 [0.02, 0.50]	1.88 [1.22, 4.47]	4 > 1	0.15 [− 0.40, 0.36]	1.01 [0.12, 4.34]	0.42 [0.10, 0.51]	0.71 [0.42, 1.96]	ns
* CYBB*	0.00 ± 0.67	2.84 ± 2.04	2.22 ± 0.44	4.73 ± 1.23	TG, CCI	0.00 ± 1.13	3.57 ± 1.69	2.46 ± 0.40	4.64 ± 1.56	TG, CCI
Glutamate pathway										
* PHGDH*	0.00 ± 0.29	− 0.20 ± 0.21	0.18 ± 0.20	0.30 ± 0.33	CCI	0.00 ± 0.30	0.05 ± 0.11	0.44 ± 0.13	0.49 ± 0.30	CCI
* GLT1*	0.00 ± 0.21	− 0.29 ± 0.24	0.09 ± 0.20	− 0.10 ± 0.35	TG	0.00 ± 0.19	− 0.08 ± 0.20	0.28 ± 0.26	0.03 ± 0.13	CCI
* GLUL*	0.00 ± 0.30	− 0.11 ± 0.26	0.12 ± 0.28	0.07 ± 0.35	ns	0.00 ± 0.24	− 0.30 ± 0.35	0.29 ± 0.32	0.15 ± 0.36	CCI
* GRIN2B*	0.00 ± 0.36	− 0.13 ± 0.40	− 0.12 ± 0.36	− 0.21 ± 0.33	ns	0.00 ± 0.42	− 0.19 ± 0.49	− 0.18 ± 0.42	− 0.40 ± 0.40	ns
* GAD1*	0.00 ± 0.26	− 0.24 ± 0.35	− 0.59 ± 0.20	− 0.65 ± 0.30	CCI	0.14 [− 0.18, 0.19]	− 0.02 [− 0.11, 0.26]	− 0.08 [− 0.63, 0.19]	− 0.62 [− 0.97, − 0.19]	ns
* GLAST*	0.00 ± 0.20	0.06 ± 0.31	0.38 ± 0.26	0.69 ± 0.26	CCI	0.00 ± 0.27	0.27 ± 0.30	0.42 ± 0.31	0.69 ± 0.31	TG, CCI
* PPARγ*	0.00 ± 0.37	− 0.23 ± 0.29	-0.88 ± 0.32	− 0.70 ± 0.57	TG, CCI	0.00 ± 0.24	− 0.28 ± 0.46	− 0.23 ± 0.36	− 0.47 ± 0.58	ns

Normally distributed data were analyzed with a 2-way ANOVA and are presented as mean ± SD. Under the ‘Significance’ column, ‘TG’ indicates a significant ANOVA main effect for *T. gondii*, ‘CCI’ indicates a significant ANOVA main effect for CCI, and ‘*’ indicates a significant ANOVA interaction between *T. gondii* and CCI. Post-hoc findings of interactive effects are detailed in the results section and Fig. 3. Non-normally distributed data were analyzed with Kruskal–Wallis tests and are presented as median [25,75 percentile]. Under the ‘Significance’ column, group differences identified with Bonferroni-corrected comparisons are summarized using the following group labels: 1 = Vehicle + Sham, 2 = *T. gondii* + Sham, 3 = Vehicle + CCI, 4 = *T. gondii* + CCI. ns = not statistically significant (i.e., *p* > 0.05)

**Table 5:** Results of gene expression analyses from the ipsilateral cortex at 18-weeks post-CCI in male and female mice

Genes	Male					Female				
	1. Vehicle + Sham (n = 5)	2. *T. gondii* + Sham (n = 5)	3. Vehicle + CCI (n = 5)	4. *T. gondii* + CCI (n = 4)	Significance	1. Vehicle + Sham (n = 5)	2. *T. gondii* + Sham (n = 4)	3. Vehicle + CCI (n = 5)	4. *T. gondii* + CCI (n = 5)	Significance
	Mean ± SD or Median [25,75 percentile]					Mean ± SD or Median [25,75 percentile]				
BBB integrity/vascular health										
* ICAM1*	0.00 ± 0.21	2.23 ± 1.00	0.85 ± 0.70	1.62 ± 0.37	TG, *	− 0.07 [− 0.14, 0.17]	1.90 [1.55, 3.07]	0.35 [0.09, 0.41]	3.29 [1.33, 3.50]	2 > 1, 4 > 1
* OCLN*	0.00 ± 0.19	0.14 ± 0.20	0.33 ± 0.18	0.19 ± 0.11	CCI	0.00 ± 0.17	0.08 ± 0.32	0.04 ± 0.16	0.00 ± 0.22	ns
* TJP1*	0.00 ± 0.12	− 0.13 ± 0.17	0.15 ± 0.18	0.05 ± 0.08	CCI	0.00 ± 0.04	0.00 ± 0.12	0.12 ± 0.10	− 0.02 ± 0.19	ns
* VEGFA*	− 0.01 [− 0.08, 0.09]	− 0.49 [− 0.56, − 0.09]	0.18 [− 0.24, 0.93]	− 0.26 [− 0.51, − 0.11]	ns	0.00 ± 0.08	− 0.26 ± 0.09	− 0.06 ± 0.30	− 0.48 ± 0.26	TG
* SYNAPTOPHYSIN*	0.05 [− 0.13, 0.11]	− 0.21 [− 0.46, − 0.09]	− 0.03 [− 0.10, 0.06]	0.00 [− 0.11, 0.14]	ns	0.00 ± 0.18	− 0.07 ± 0.22	0.01 ± 0.11	− 0.28 ± 0.19	TG
* HIF1A*	0.00 ± 0.16	− 0.18 ± 0.28	− 0.08 ± 0.14	0.06 ± 0.20	ns	0.00 ± 0.17	0.12 ± 0.13	0.07 ± 0.12	0.21 ± 0.32	ns
* MAP2*	0.00 ± 0.09	− 0.27 ± 0.28	− 0.09 ± 0.23	− 0.08 ± 0.03	TG, CCI	0.00 ± 0.11	− 0.03 ± 0.06	0.01 ± 0.14	− 0.35 ± 0.12	TG, CCI, *

Normally distributed data were analyzed with a 2-way ANOVA and are presented as mean ± SD. Under the ‘Significance’ column, ‘TG’ indicates a significant ANOVA main effect for *T. gondii*, ‘CCI’ indicates a significant ANOVA main effect for CCI, and ‘*’ indicates a significant ANOVA interaction between *T. gondii* and CCI. Post-hoc findings of interactive effects are detailed in the results section and Fig. 3. Non-normally distributed data were analyzed with Kruskal–Wallis tests and are presented as median [25,75 percentile]. Under the ‘Significance’ column, group differences identified with Bonferroni-corrected comparisons are summarized using the following group labels: 1 = Vehicle + Sham, 2 = *T. gondii* + Sham, 3 = Vehicle + CCI, 4 = *T. gondii* + CCI. ns = not statistically significant (i.e., *p* > 0.05)

#### 2-h post-injury

With regards to interactive/synergistic effects of *T. gondii* and TBI at 2-h post-injury in males, Kruskal–Wallis tests found that only the *T. gondii* + CCI mice had increased expression of the inflammatory cytokine *IFNγ* (Fig. 3A) compared to both the Vehicle + CCI and Vehicle + Sham mice (*p* < 0.05). On the measure of the inflammatory chemokine *CXCL10* (Fig. 3B)*,* two-way ANOVA found a significant *T. gondii* * CCI interaction (F_1,18_ = 4.91, *p* = 0.040)*.* Bonferroni post-hoc comparisons indicated that while *T. gondii* + CCI, *T. gondii* + Sham, and Vehicle + CCI mice all had increased *CXCL10* compared to Vehicle + Sham, the *T. gondii* + CCI mice were the only group that also had increased *CXCL10* than the Vehicle + CCI mice (*p* < 0.05). On the measures of the inflammatory cytokines *IL6* (Fig. 3C) and *IL10* (Fig. 3D), Kruskal–Wallis tests found that only the *T. gondii* + CCI mice had elevated expression compared to the Vehicle + Sham mice (*p* < 0.05).

In the females, Kruskal–Wallis tests found that only the *T. gondii* + CCI mice had increased expression of the inflammatory cytokine *IL2* (Fig. 3E) compared to both the Vehicle + CCI and Vehicle + Sham mice (*p* < 0.05). On the measures of *IFNγ* (Fig. 3F), *SOCS1* (Fig. 3G), *GFAP* (Fig. 3H), and *FOXP3* (Fig. 3I), Kruskal–Wallis tests found that while both *T. gondii* + CCI and *T. gondii* + Sham mice had elevated expression compared to the Vehicle + Sham mice (*p* < 0.05), only the *T. gondii* + CCI group had increased expression than the Vehicle + CCI mice (*p* < 0.05). On the measure of *CCL4* (Fig. 3J), Kruskal–Wallis test found that while both *T. gondii* + CCI and Vehicle + CCI mice had elevated *CCL4* compared to the Vehicle + Sham mice (*p* < 0.05), only the *T. gondii* + CCI group had increased expression than the *T. gondii* + Sham mice (*p* < 0.05). On the measures of *CCL2* (Fig. 3K), *IL12p40* (Fig. 3L), and *ARG1*(Fig. 3M), Kruskal–Wallis tests found that only the *T. gondii* + CCI mice had elevated expression compared to the Vehicle + Sham mice (*p* < 0.05). On the measure of *CXCR3* (Fig. 3N), Kruskal–Wallis tests found that while both *T. gondii* + CCI and *T. gondii* + Sham mice had elevated expression compared to the Vehicle + Sham mice (*p* < 0.05), only the *T. gondii* + Sham group had increased expression than the Vehicle + CCI mice (*p* < 0.05). Finally, on the measures of *CXCL10* and *CCL12,* two-way ANOVA found a significant *T. gondii* * CCI interaction (*CXCL10:* F_1,20_ = 4.94, *p* = 0.038, Fig. 3O; *CCL12:* F_1,20_ = 5.64, *p* = 0.028, Fig. 3P). Post-hoc comparisons found that *T. gondii* + CCI, *T. gondii* + Sham, and Vehicle + CCI mice all had increased *CXCL10* compared to Vehicle + Sham, while the *T. gondii* + CCI and *T. gondii* + Sham also had increased levels compared to the Vehicle + CCI mice (*p* < 0.05).

#### 24-h post-injury

**Fig. 4:**
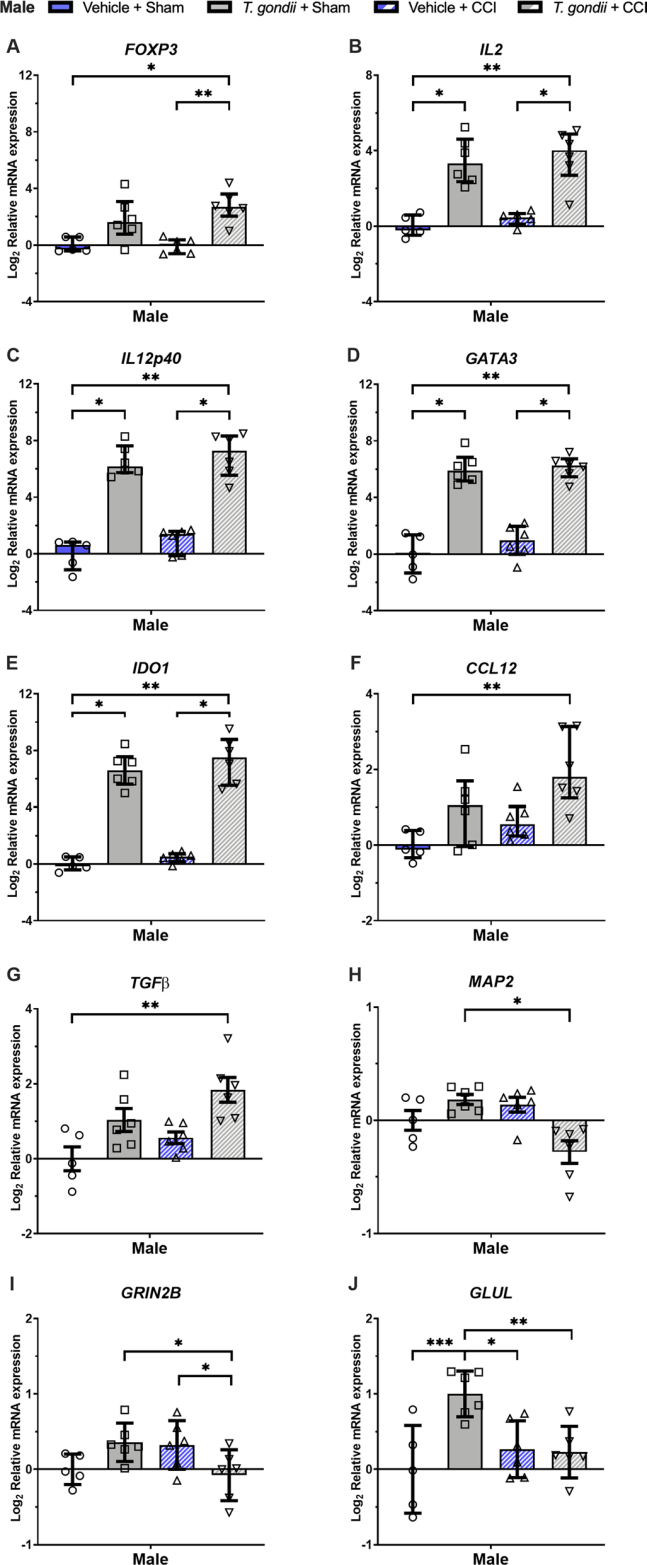
Gene expression findings from the ipsilateral cortex suggesting interactive effects of *T. gondii* and CCI at 24-h post-injury in male mice. Relative to *T. gondii* and/or CCI in isolation, male mice with combined *T. Gondii* and CCI had evidence of altered gene expression related to neuroinflammation (**A–D, F, G**), oxidative stress **(E)**, neuronal integrity **(H)**, and glutamatergic pathways **(I, J)**. **I and J** were analyzed with 2-way ANOVA and the significant *T. Gondii* * CCI interaction was further analyzed with Bonferroni post-hoc comparisons. These normally distributed data are displayed as mean ± SD. Data in **A–H** were not normally distributed and were therefore analyzed with Kruskal–Wallis tests and presented as median with interquartile range. *p < 0.05, **p ≤ 0.01. ***p ≤ 0.001

In males, Kruskal–Wallis tests found that only the *T. gondii* + CCI mice had increased expression of *FOXP3* (Fig. 4A) compared to both the Vehicle + CCI and Vehicle + Sham mice (*p* < 0.05). On the measures of *IL2* (Fig. 4B), *IL12p40* (Fig. 4C), *GATA3* (Fig. 4D), and *IDO1* (Fig. 4E), Kruskal–Wallis tests found that while both *T. gondii* + CCI and *T. gondii* + Sham mice had elevated expression compared to the Vehicle + Sham mice (*p* < 0.05), only the *T. gondii* + CCI group had increased expression than the Vehicle + CCI mice (*p* < 0.05). Kruskal–Wallis tests also found that only the *T. gondii* + CCI mice had elevated *CCL12* (Fig. 4F) and TGFβ (Fig. 4G) compared to the Vehicle + Sham mice (*p* < 0.05), and decreased *MAP2* (Fig. 4H) expression than *T. gondii* + Sham mice (*p* < 0.05). Two-way ANOVA found significant T. gondii*CCI interactions for *GRIN2B* (F_1,19_ = 9.91, *p* = 0.005; Fig. 4I) and *GLUL* (F_1,19_ = 9.39, *p* = 0.006; Fig. 4J). Post-hoc analysis found that *T. gondii* + CCI had lower *GRIN2B* expression than *T. gondii* + Sham and Vehicle + CCI mice (*p* < 0.05), and that *T. gondii* + Sham had higher *GLUL* expression than all groups (*p* < 0.05).

**Fig. 5:**
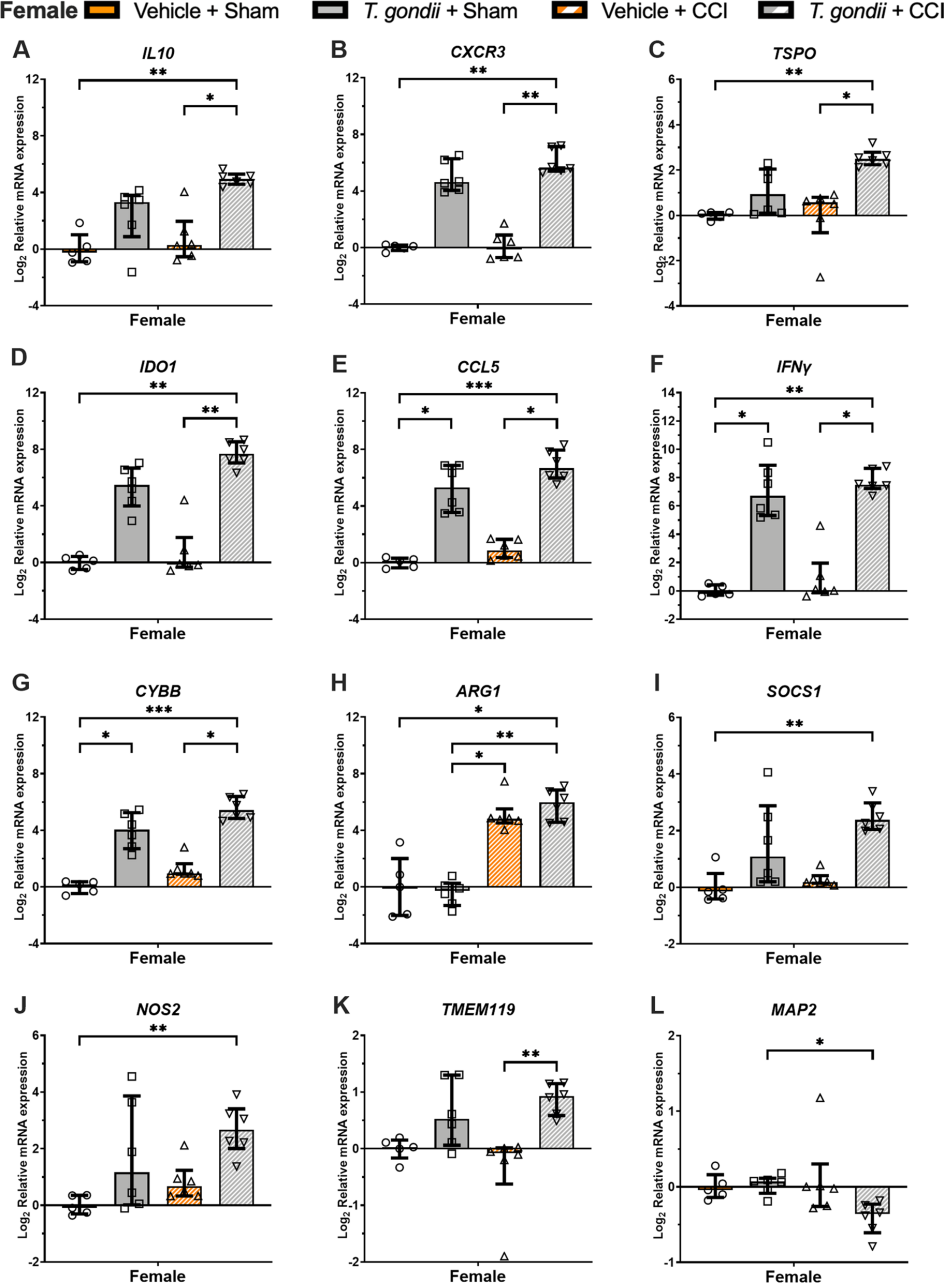
Gene expression findings from the ipsilateral cortex suggesting interactive effects of *T.gondii* and CCI at 24-h post-injury in female mice. Relative to *T. gondii* and/or CCI in isolation, female mice with combined *T. Gondii* and CCI had evidence of altered gene expression related to neuroinflammation (**A–C, F, H, I, K),** oxidative stress **(D, G, J),** and neuronal integrity **(L)**. Data were not normally distributed and were analyzed with Kruskal–Wallis tests. Data presented as median with interquartile range. *p < 0.05, **p ≤ 0.01. ***p ≤ 0.001

In females, Kruskal–Wallis tests found that only the *T. gondii* + CCI mice had increased expression of *IL10* (Fig. 5A), *CXCR3* Fig. 5B), *TSPO* (Fig. 5C), and *IDO1* (Fig. 5D) compared to both the Vehicle + CCI and Vehicle + Sham mice (*p* < 0.05). On the measures of *CCL5* (Fig. 5E), *IFNγ* (Fig. 5F) and *CYBB* (Fig. 5G), Kruskal–Wallis tests found that while both *T. gondii* + CCI and *T. gondii* + Sham mice had elevated expression compared to the Vehicle + Sham mice (*p* < 0.05), only the *T. gondii* + CCI group had increased expression than the Vehicle + CCI mice (*p* < 0.05). On the measure of *ARG1* (Fig. 5H), Kruskal–Wallis test found that while both *T. gondii* + CCI and Vehicle + CCI mice had elevated *ARG1* compared to the Vehicle + Sham mice (*p* < 0.05), only the *T. gondii* + CCI group had increased expression than the *T. gondii* + Sham mice (*p* < 0.05). On the measures of *SOCS1* (Fig. 5I) and *NOS2* (Fig. 5J), Kruskal–Wallis tests found that only the *T. gondii* + CCI mice had elevated expression compared to the Vehicle + Sham mice (*p* < 0.05). Finally, Kruskal–Wallis tests also found that only the *T. gondii* + CCI mice had elevated expression of *TMEM119* compared to the Vehicle + CCI mice (Fig. 5K, p < 0.05), and decreased *MAP2* compared to the *T. gondii* + Sham mice (Fig. 5L, p < 0.05).

#### 7-days and 18-weeks post-injury

**Fig. 6:**
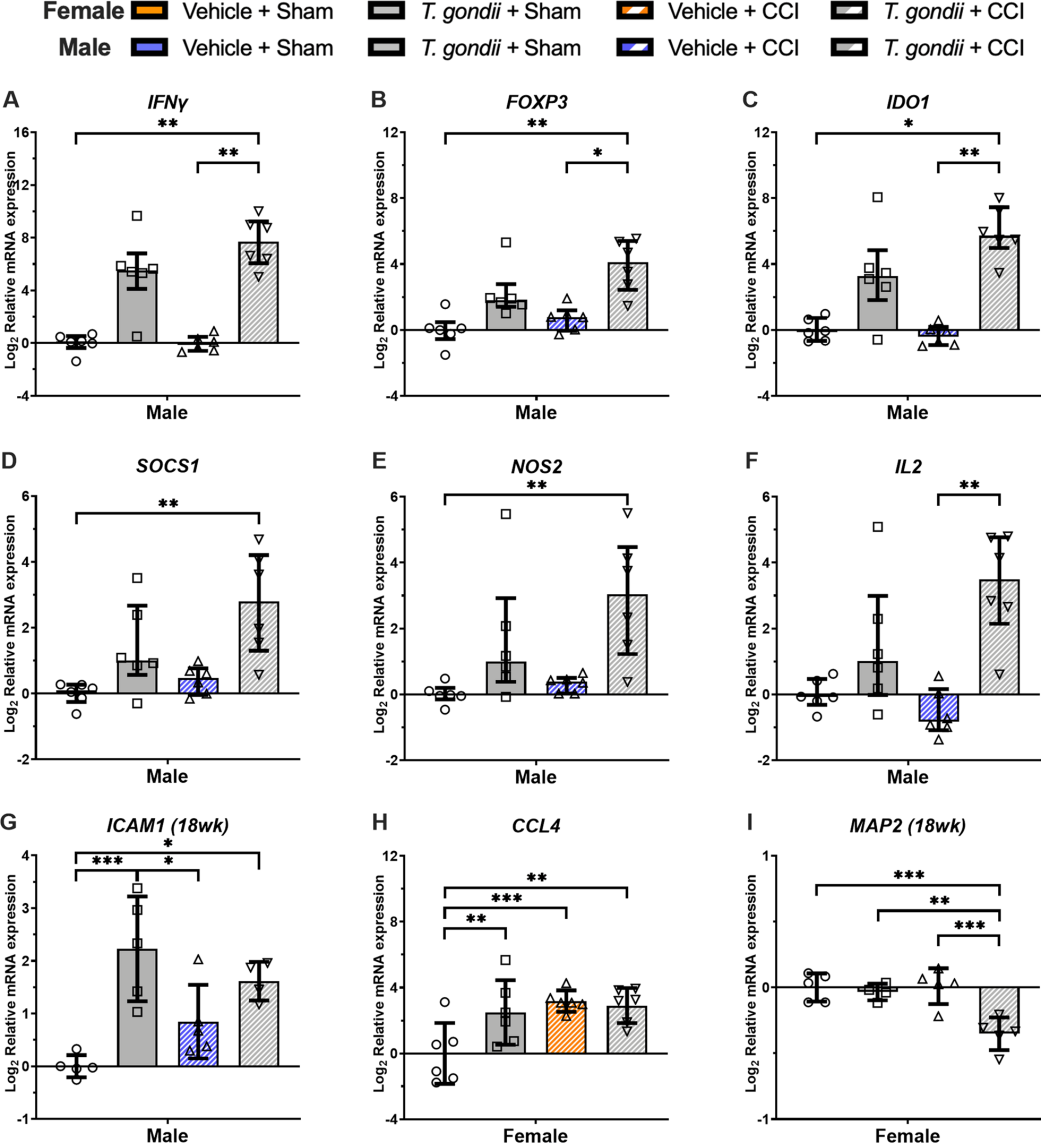
Gene expression findings from the ipsilateral cortex suggesting interactive effects of *T.gondii* and CCI at 7-days and 18-weeks post-injury in male and female mice. Relative to *T. gondii* and/or CCI in isolation, male mice with combined *T. gondii* and CCI had evidence of altered gene expression related to neuroinflammation (**A, B, D, F)** and oxidative stress **(C, E)** at 7-days post-CCI, as well as *ICAM1* at 18-weeks post-injury **(G)**. In females, there was a significant *T. Gondii* * CCI interaction indicating that all groups had elevated gene expression of *CCL4* when compared to Vehicle + Sham mice at 7-days post-injury **(H)**, and the *T. gondii* + CCI group had decreased expression of *MAP2* than all other groups at 18-weeks post-injury **(I)**. **G–I** were analyzed with 2-way ANOVA and the significant *T. Gondii* * CCI interaction was further analyzed with Bonferroni post-hoc comparisons. These normally distributed data are displayed as mean ± SD. Data in **A–F** were not normally distributed and were therefore analyzed with Kruskal–Wallis tests and presented as median with interquartile range. *p < 0.05, **p ≤ 0.01. ***p ≤ 0.001

At 7-days post-injury in males, Kruskal–Wallis tests found that only the *T. gondii* + CCI mice had increased expression of *IFNγ* (Fig. 6A), *FOXP3* (Fig. 6B), and *IDO1* (Fig. 6C) compared to both the Vehicle + CCI and Vehicle + Sham mice (*p* < 0.05). On the measures of *SOCS1* (Fig. 6D) and *NOS2* (Fig. 6E), Kruskal–Wallis tests found that only the *T. gondii* + CCI mice had elevated expression compared to the Vehicle + Sham mice (*p* < 0.05). On the measure of *IL2* (Fig. 6F), Kruskal–Wallis tests found that only the *T. gondii* + CCI mice had elevated expression compared to the Vehicle + CCI mice (*p* < 0.05). At 18-weeks post-injury in the males, two-way ANOVA found a significant *T. gondii* * CCI interaction for *ICAM1* (F_1,15_ = 5.77, *p* = 0.030; Fig. 6G). Post-hoc analysis found that while both *T. gondii* + CCI and *T. gondii* + Sham mice had elevated *ICAM1* compared to the Vehicle + Sham mice (*p* < 0.05), only the *T. gondii* + Sham group had increased expression than the Vehicle + CCI mice (*p* < 0.05).

In females at 7-days post-injury, two-way ANOVA found a significant *T. gondii* * CCI interaction for *CCL4* expression (F_1,20_ = 5.19, *p* = 0.034; Fig. 6H), and post-hoc analysis found that Vehicle + Sham had lower levels of *CCL4* expression compared to all other groups (*p* < 0.05). At 18 weeks, two-way ANOVA found a significant T. gondii*CCI interaction for *MAP2* expression (F_1,15_ = 9.74, *p* = 0.007; Fig. 6I), with post-hoc analysis finding that *T. gondii* + CCI had lower expression of *MAP2* than all other groups (*p* < 0.05).

### A pre-existing *T. gondii* infection exacerbates chronic TBI lesion size in males

**Fig. 7:**
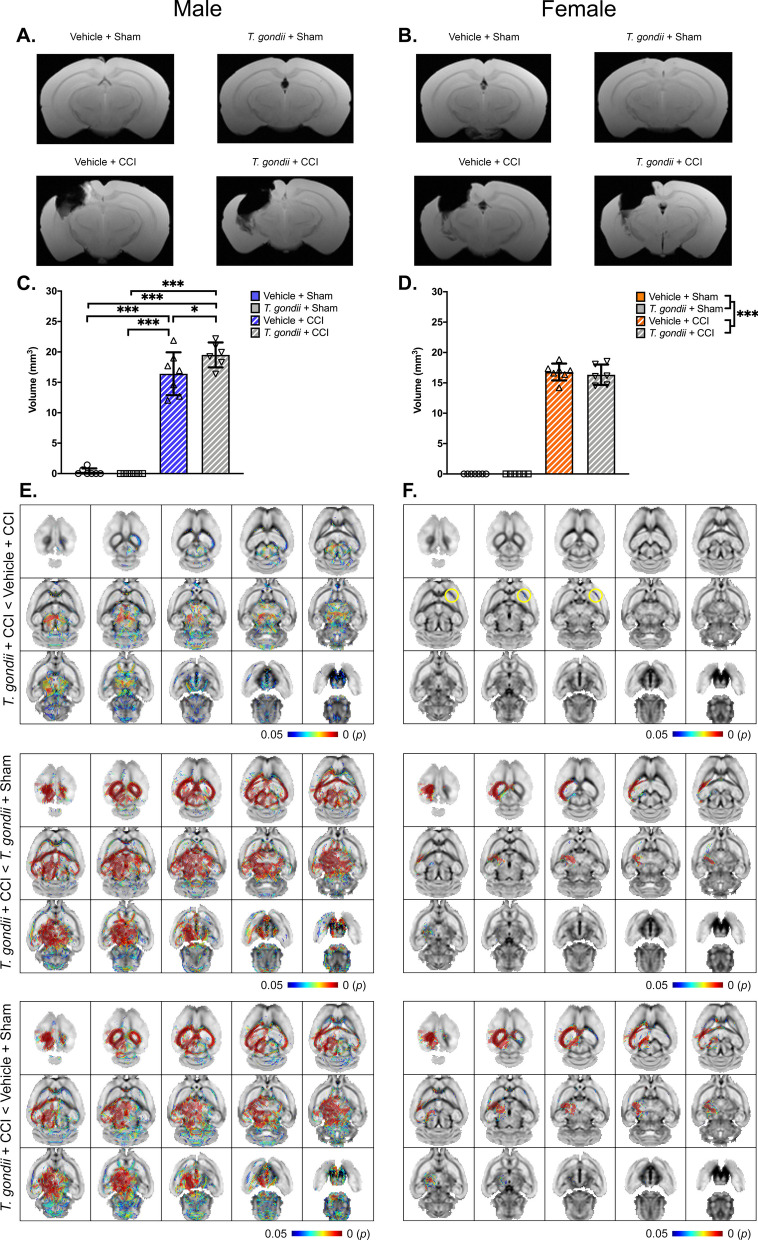
MRI analysis of lesion volume and white matter tract abnormalities 18-weeks post- injury. 3D multi-gradient echo images were acquired through ex-vivo MRI for male **(A)** and female **(B)** mice to assess lesion size in the ipsilateral cortex. In males, the CCI groups had larger lesions than sham groups; however, the *T. gondii* + CCI mice also had a larger lesion volume than the Vehicle + CCI mice **(C)**. In females, CCI mice had larger lesions than sham-injured groups but, unlike males, no difference was found between *T. gondii* + CCI and Vehicle + CCI groups **(D)**. Whole-brain statistical testing with connectivity based fixel enhancement revealed that *T. gondii* + CCI males had white matter tract abnormalities that spanned both hemispheres when compared to Vehicle + CCI males **(E)**. In females, more subtle differences in the contralateral corpus callosum were observed between *T. gondii* + CCI and Vehicle + CCI groups **(F)**. Data displayed as mean ± SD. *p < 0.05, ***p ≤ 0.001. n = 6–7/ group/ sex

MRI was used to measure lesion size at 18-weeks post-injury in male (Fig. 7A) and female mice (Fig. 7B). In males, two-way ANOVA found a main effect of CCI (F _(1,23)_ = 503.65, *p* < 0.001) and a significant *T. gondii* * CCI interaction (F _(1,23)_ = 4.56, *p* = 0.044; Fig. 7C). Post-hoc analyses found that *T. gondii* + CCI mice had larger lesions compared to all other groups (*p* < 0.05). *T. gondii* + CCI males also had increased lesion volume compared to both sham groups (*p* < 0.001). In females, a main effect of injury was evident (F _(1,22)_ = 1700.81, *p* < 0.001; Fig. 7D); however, there was no difference between the *T. gondii* + CCI females (i.e., no significant *T. gondii* * CCI interaction).

### A pre-existing *T. gondii* infection exacerbates chronic white matter abnormalities after TBI, and this is more pronounced in males

White matter connectivity changes on diffusion MRI were assessed using connectivity-based fixel enhancement. When compared to the other male groups, the male *T. gondii* + CCI mice had widespread ipsilateral and contralateral abnormalities in corpus callosum, corticospinal tract, anterior commissure, fimbria, cerebellum, hippocampus, striatum, pallidum, superior colliculus, thalamus, and hypothalamus fiber bundles (Fig. 7E). Although female *T. gondii* + CCI mice also had white matter tract abnormalities in comparison to Vehicle + CCI females (Fig. 7F; significant differences highlighted by yellow circles), this was more subtle than what occurred in the males and focused in the contralateral corpus callosum. When compared to the *T. gondii* + Sham and Vehicle + Sham females, the *T. gondii* + CCI females had changes in white matter connectivity in the ipsilateral hemisphere including corpus callosum, fimbria, corticospinal tract hippocampus, striatum, and thalamus fiber bundles.

### A pre-existing *T. gondii* infection did not worsen long-term behavioral deficits following TBI

**Fig. 8:**
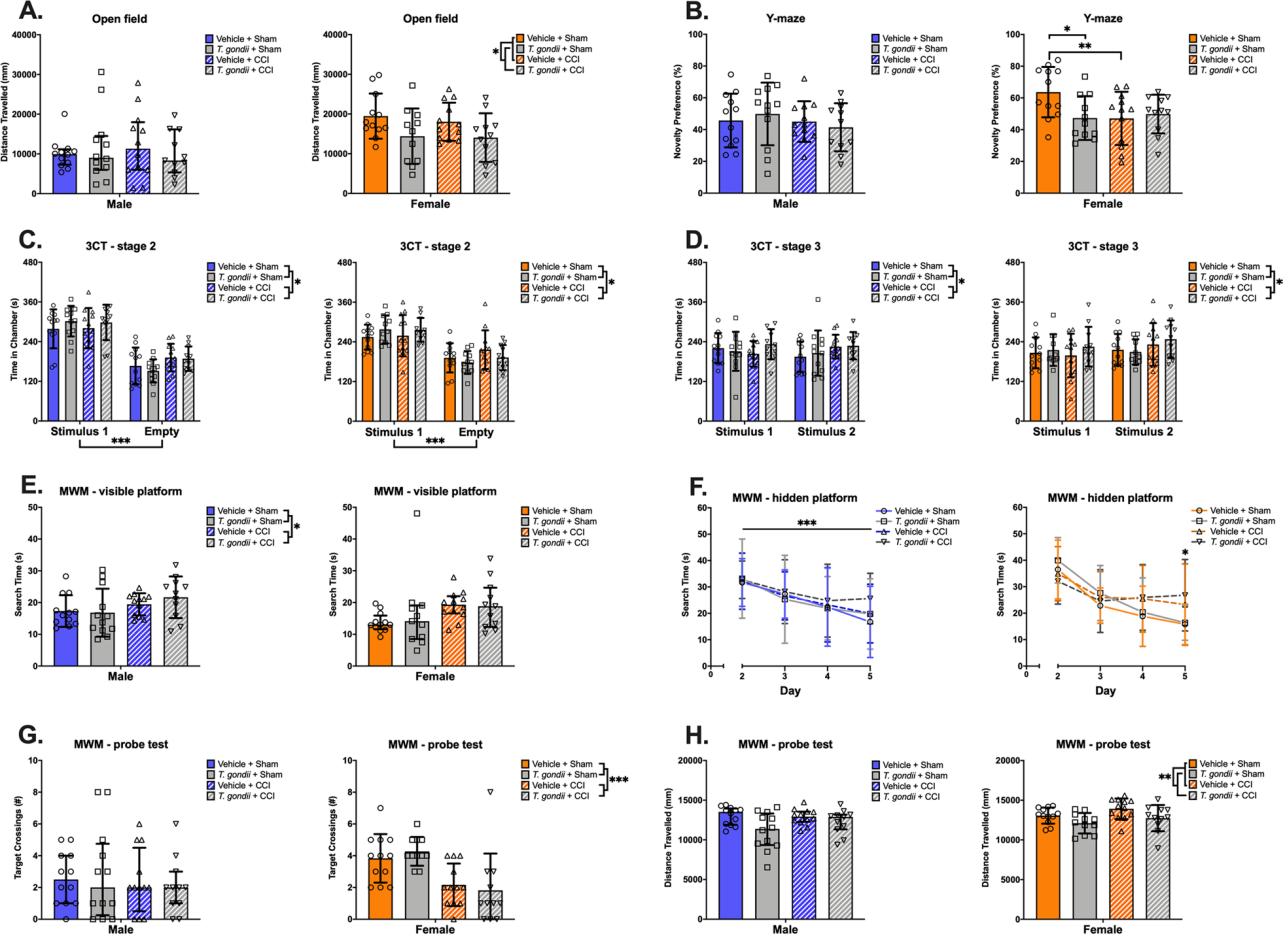
Behavioral findings of sex-specific main effects for *T. gondii* or CCI. *T. gondii* female mice travelled less in the open field compared to vehicle females **(A)**. Vehicle + CCI and *T. gondii* + Sham females had less of a preference for the novel arm in the Y-maze compared to Vehicle + Sham females **(B)**. Stage 2 of the 3-chamber social test (3CT) revealed a preference for the ‘stimulus 1’ chamber in both sexes, and CCI mice spent more time in the side chambers **(C)**. During stage 3 of the 3CT, neither male nor female mice had a preference for the unfamiliar ‘stimulus 2’ mouse, while CCI mice again spent more time in the side chambers **(D)**. In the water maze, CCI males took longer on average to find the visible platform **(E)**, and female CCI mice took longer on day 5 of the task to locate the hidden platform **(F)**. On the probe test, CCI females made fewer crosses through the target platform location compared to sham-injured females **(G)**. *T. gondii* females travelled less in the probe test compared to Vehicle mice **(H)**. Data displayed as mean ± SD when analyzed with 2-Way ANOVA or as median with interquartile range when analyzed with Kruskal–Wallis. *p < 0.05, **p ≤ 0.01. ***p ≤ 0.001. n = 11–12/group/sex

In the open field (Fig. 8A), locomotion was impacted in female *T. gondii* mice only, such that *T. gondii* infected females showed a decrease in distance moved compared to vehicle mice (F _(1,42)_ = 6.68, *p* = 0.013). Locomotion was comparable between male groups, and no significant differences in distance travelled were detected.

In the Y-maze (Fig. 8B), a significant infection*injury interaction on novelty preference in female mice was observed (F _(1,42)_ = 4.73, *p* = 0.035). *Post-hoc* analyses revealed that both Vehicle + CCI and *T. gondii* + Sham females had less of a preference for the novel arm compared to Vehicle + Sham female mice (*p* = 0.009 and *p* = 0.012, respectively). No differences in novelty preference were found between male groups.

During the second stage of the 3CT (Fig. 8C), which is used to assess sociability and social exploration, a main effect of chamber and injury were found on the time spent in the side chambers in males (F _(1,43)_ < 0.001, *p* = and F _(1,43)_ = 7.00, *p* = 0.011, respectively) and females (F _(1,42)_ = 31.76, *p* < 0.001 and F _(1,42)_ = 6.35, *p* = 0.016, respectively). In males and females, mice tended to spend more time in the ‘stimulus 1’ mouse chamber compared to the empty chamber. During the third stage of the 3CT (Fig. 8D), which is used to assess social memory and preference, a main effect of injury was found on the time spent in the ‘stimulus 1’ and ‘stimulus 2’ chambers in males (F _(1,43)_ = 4.31, *p* = 0.044) and females (F _(1,42)_ = 4.17, *p* = 0.047). Here, CCI male and female mice spent more time overall within the side chambers compared to sham-injured mice. However, neither male or female groups had a social preference for the unfamiliar ‘stimulus 2’ mouse compared to the ‘stimulus 1’ mouse, and mice of both sexes spent a similar amount of time across the two social chambers.

The water maze (Fig. 8E–H) was completed across three consecutive stages: the visible platform, the hidden platform, and the probe test. During the visible platform stage, CCI males took longer to find the platform compared to sham-injured male mice, indicating impaired spatial learning (F _(1,43)_ = 4.18, *p* = 0.047; Fig. 8E). Although a difference in search time was also detected between female groups (H _(3)_ = 8.03, *p* = 0.045), no significant multiple comparisons were found. During the hidden platform stage, a main effect of day was observed in males, such that male mice were able to learn the task and spend less time to find the platform across the four days (F _(3,129)_ = 13.24, *p* < 0.001; Fig. 8F). In females, a main effect of day and a significant day*injury interaction was observed (F _(3,126)_ = 31.26, *p* < 0.001 and F _(3,126)_ = 6.36, *p* < 0.001; Fig. 8F). *Post-hoc* analyses found that CCI female mice spent more time searching for the platform on day 5 of the task compared to sham-injured mice (*p* = 0.012). Also, CCI females had significantly reduced search time between days 2 to 3 (*p* = 0.011) and days 2 to 5 (*p* = 0.011), but not between day 3 to 5 of the task. On the other hand, vehicle females had significantly reduced search time between days 2 to 3 (*p* < 0.001), 2 to 4 (*p* < 0.001), 2 to 5 (*p* < 0.001), 3 to 4 (*p* = 0.043) and 3 to 5 (*p* = 0.001). Overall, this suggests impaired spatial learning and memory in CCI females compared to sham-injured females. During the probe test, males made a similar number of target platform crossings irrespective of group (Fig. 8G). On the other hand, CCI females crossed the target platform location fewer times compared to sham-injured mice (F _(1,42)_ = 19.10, *p* < 0.001), again indicating impaired spatial memory in females. In addition, in females a main effect of infection on distance travelled in the probe task was evident (F _(1,42)_ = 7.37, *p* = 0.010; Fig. 8H), by which *T. gondii*-infected females travelled less compared to Vehicle mice. No differences in distance travelled was observed between male groups.

Additional behavioral measures assessed showed limited independent effects of infection and injury and are summarized in Additional file 2: Figures S1-S3.

## Discussion

**Fig. 9:**
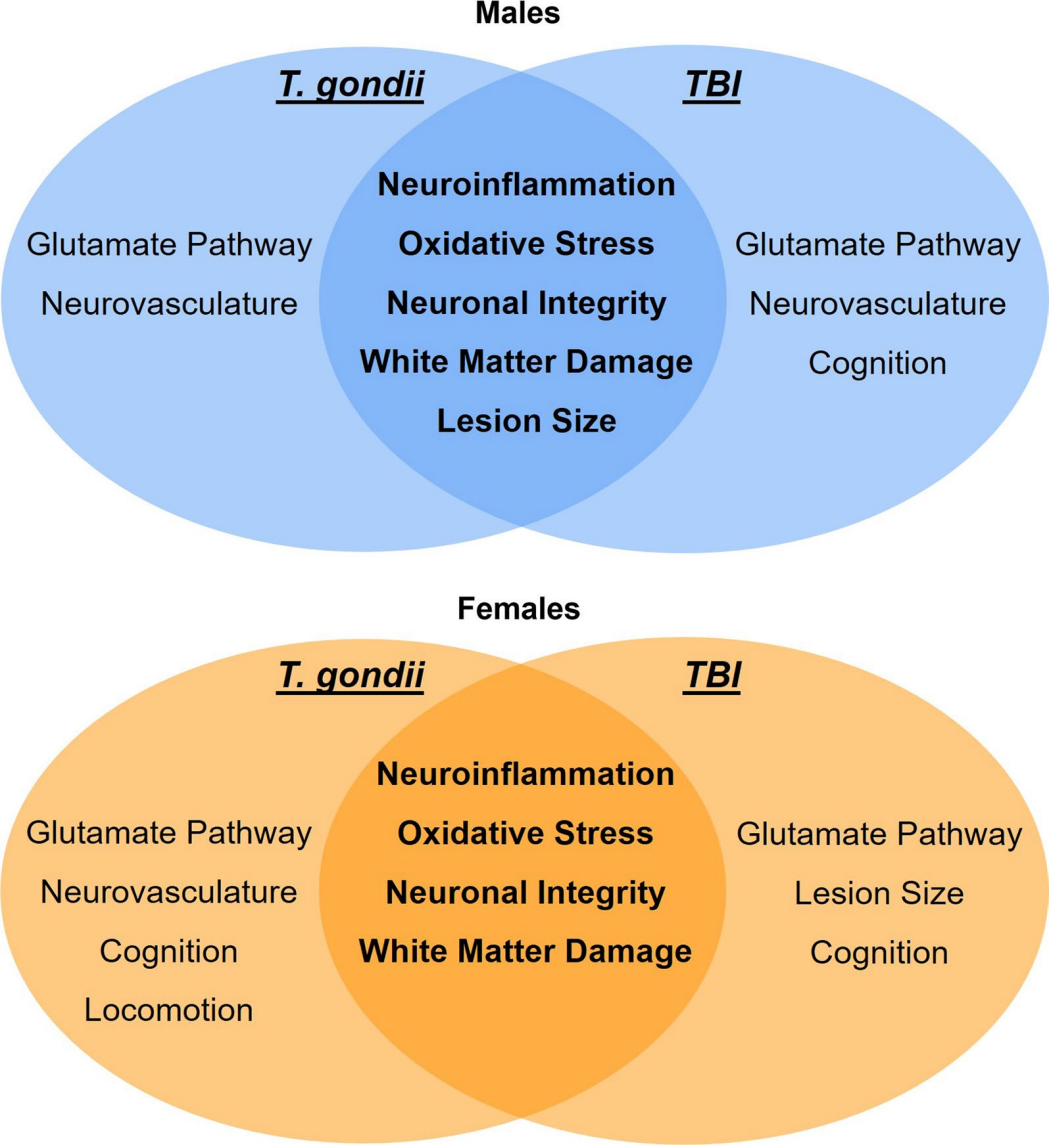
Summary of findings. Main effects related to *T. gondii* (left), main effects related to TBI (right), and synergistic/interactive effects of *T. gondii* + TBI (center) in males (top, blue) and females (bottom, orange)

*T. gondii* is commonly acquired via foodborne transmission and thereby must disseminate from the gastrointestinal system of its host and breach the BBB where it then permanently resides within the central nervous system (CNS) [33, 37, 40]. In response to this, is well established that resident CNS cells (i.e., microglia and astrocytes) become activated and increase in abundance while peripheral immune cells (e.g., macrophages, natural killer cells and T-lymphocytes) are recruited across the BBB to assist in parasite control [41]. As such, in the context of TBI, a pre-existing chronic *T. gondii* infection should result in the brain already being in an inflamed state at the time of TBI which could have important implications on the consequent pathobiology and recovery. In this study we therefore examined the effects of a pre-existing *T. gondii* infection on the acute, sub-acute, and chronic aftermath of TBI in male and female mice. We found that a pre-existing *T. gondii* infection does exacerbate some of the neuropathophysiological outcomes after TBI, including an intensified early immune response and more severe chronic brain damage (see summary of main findings in Fig. 9). While some of these effects were relatively consistent in both sexes, other findings, such as the exacerbated brain damage on MRI outcomes being more severe in males, appeared to be influenced by sex. In addition to these interactive effects, independent effects of both *T. gondii* infection and TBI were also evident at the molecular, structural, and functional level, providing further understanding of the short- and long-term consequences of these conditions in both sexes.

Gene expression analyses at 2-h, 24-h, and 1-week post-injury found that in both sexes a pre-existing *T. gondii* infection increased the expression of numerous pro- and anti-inflammatory mediators; increased the expression of markers reflective of activated microglia and macrophages (*i.e. IBA1, TMEM119, CD86)*, astrocytes (*GFAP)*, T-lymphocyte transcription factors (i.e., *GATA3, STAT1, FOXP3*), and T-lymphocyte surface markers (i.e., *CXCR3*); and altered the expression of glutamatergic, neurotoxic, and oxidative stress mediators. Overall, these gene expression findings align with current knowledge of how a chronic *T. gondii* infection is maintained via activation of the host immune system [41, 44, 68], as do the findings in the VEH + CCI mice demonstrating that a TBI induces an acute and sub-acute neuroinflammatory response [12, 13, 44]. However, in effect, the immune processes involved in *T. gondii* infection control appear to shift the consequent neuroinflammatory profile during acute and sub-acute TBI recovery in both male and female mice. These broad neuroinflammatory alterations may be attributed to the characteristic CD4^+^ T-helper 1 response, resultant IFNγ production and IFNγ-mediated activation of macrophages, and CD8^+^ T-cell mediated cytotoxicity, which are critical mechanisms for *T. gondii* infection control [69]. In this study, expression of *IFNγ* was increased in *T. gondii* + CCI mice during the acute and sub-acute recovery. IFNγ is known to induce CXCL10 and IDO1 production [70, 71], and our results indicate that *CXCL10, CXCR3,* and *IDO1* expression were also increased in *T. gondii* and CCI conditions. Considering that activation of the CXCR3/ CXCL10 axis can enhance neuronal excitability [72], and that IDO1 production can favor tryptophan degradation to neurotoxic metabolites such as quinolinic acid [73], it is also plausible that these early but sustained processes, among others, may have contributed to increased excitotoxicity and secondary damage. It should also be noted that the expression of *GLUL* (encodes for glutamine synthetase; an enzyme that catalyzes the production of glutamine from glutamate) was significantly increased in male *T. gondii-*infected sham mice at 24-h compared to all other groups. Taken together with increased *GLAST* (encodes for the excitatory amino acid transporter GLAST; located exclusively on astrocytes) and decreased *GAD1* (encodes for glutamate decarboxylase 1; an enzyme that catalyzes the production of gamma-aminobutyric acid (GABA)) in *T. gondii-*infected males at this timepoint, it is plausible that high levels of extracellular glutamate were evident in male *T. gondii-*infected sham mice, and compensatory pathways to catabolize and uptake glutamate were needed. On the other hand, *GLUL* was decreased in female Vehicle + CCI and *T. gondii* + CCI mice compared to sham-injured females, and perhaps the conversion of glutamate to GABA, or to the citric acid cycle was favored in female mice. However, due to the associative nature of our study, future investigations are required to better understand the relationship between the various pathophysiological outcomes in this study.

Ex-vivo MRI found that in a combined *T. gondii* + TBI setting, both males and females had evidence of exacerbated chronic brain injury compared to either *T. gondii* or TBI alone. This was particularly prominent in the *T. gondii* + CCI male mice, who had increased lesion volume in addition to white matter abnormalities across multiple fiber bundles at 18-weeks post-injury compared to the mice that only had TBI. Subtle white matter abnormalities in the corpus callosum were also evident in females in the combined *T. gondii* + TBI group compared to the TBI only group; however, no difference in lesion volume was observed. These findings are somewhat counterintuitive to the decrease in *MAP2* (i.e., plays a key function in microtubule assembly and stabilization of neurons) gene expression in the ipsilateral cortex of female, but not male, *T. gondii* + CCI mice at 18-weeks post-injury. It is possible that these molecular changes in female *T. gondii* + CCI mice precede future degeneration/increased lesion volume, although additional studies are required to investigate this as well as the relationship between *MAP2* gene expression and macroscopic imaging outcomes.

Despite evidence that the combined *T. gondii* + CCI resulted in worse pathophysiology and long-term brain damage, there were no synergistic effects on long-term behavior outcomes. As demonstrated by our MRI lesion findings, the CCI model results in considerable brain damage regardless of a concomitant *T. gondii* infection. On the other hand, at the time points examined, CCI mice had only minor behavioral abnormalities that do not align with or reflect the degree of brain damage present. Considering the lack of behavioral deficits in CCI versus sham mice (i.e., where there is a large difference in the extent of brain damage), it is therefore unsurprising that the *T. gondii* + CCI and Vehicle + CCI (i.e., where there is a relatively smaller difference in the extent of brain damage) did not differ on behavior. This is consistent with previous mouse studies from our laboratory involving other concomitant insults (e.g., TBI + fracture) that demonstrated altered pathophysiology and exacerbated brain damage compared to isolated TBI, but no manifestation of behavioral differences [74]. The mismatch between brain damage and behavior findings could be due to numerous factors including poor sensitivity of the behavior tests used, as well as the brain’s compensatory mechanisms; both of which require future research.

There are some limitations to consider when interpreting these findings. For example, our pathophysiological studies were limited to gene expression analysis, and it would have been informative to conduct an analysis of protein expression as well as immunohistological investigations into the spatial profiles and phenotypes of different cell types. Our gene analysis was also restricted to the ipsilateral cortex, which was chosen because it is the location of the CCI impact and likely to have pathophysiological changes within it. However, future studies should analyze other brain regions including the white matter—especially considering our diffusion MRI findings of white matter abnormalities and previous evidence indicating that systemic infection and TBI can both result in inflammatory-mediated white matter damage [75–78]. Our analysis was also limited because it did not include recovery time as a factor due to each recovery time consisting of rats from different litters/cohorts. We also employed a model of moderate-to-severe TBI, and it would be interesting to expand this research to encompass mild TBI; especially considering the high prevalence of mild TBI and recent findings that other forms of infection (e.g., cytomegalovirus) contribute to brain structure abnormalities after concussion [79]. Further studies investigating the timing of infection and TBI (e.g., pre- versus post-TBI, chronic versus acute) would also be clinically relevant and could influence results. In addition, we did not measure the burden of the parasite in the brain. Although this has been reported in previous studies that have used the same mouse strain and *T. gondii* model as the current study [80], further analysis of region-specific parasite burden in the brain could aid in the interpretation of the findings in this study. The role of the brain-periphery axis in our findings offers another topic for future investigation. *T. gondii* was delivered via i.p. injection and a systemic immune response was evident based on the spleen weight findings. Of note, chronic *T. gondii* infection has been found to cause lipofuscinosis, collagenopathy, splenic T-cell dysfunction and spleen and white pulp atrophy [56, 81]. At the acute and sub-acute recovery times, male and female *T. gondii* mice had increased expression of genes reflective of macrophages and lymphocytes in the brain, suggesting infiltration of peripheral immune cells that coincided with increased spleen weights. While many of these changes occurred independent of whether the animal also received a TBI, there was some evidence that this was exacerbated in the combined *T. gondii* + CCI setting. At the chronic 18-week recovery time, only the male *T. gondii* mice continued to have a statistically significant increase in spleen weight; however, previous findings from our laboratory show that both the male and female *T. gondii* infected mice have increased expression of genes related to leukocytes. Taken together, further studies are required to investigate the relationship between histopathological splenic changes, circulating immune cells, and the neuroimmune response in chronic T. gondii infection both with and without a concomitant TBI. The use of sulfadiazine therapy to prevent mortality during the acute stage of *T. gondii* infection is another possible limitation and may have inadvertently altered the cyst burden and degree of neuroinflammation in the chronic infection setting, and previous studies indicate that this is dependent on the sulfadiazine concentration, length of therapy, and time and route of delivery [82–84]. Any confounding effects of the sulfadiazine therapy in the present study were likely negligible because the treatment was given to both the uninfected and infected mice, and the treatment regimen used was relatively minimal compared to the previous studies showing chronic alterations in infection pathophysiology in similar mice and *T. gondii* strains. Finally, it is important that these findings be validated in human studies. This might involve retrospective analyses of current datasets (e.g., if infection status data exists or if there are samples that permit serologic testing) or new prospective studies that screen for *T. gondii* infection in acute TBI patients who are then studied longitudinally. It would also be interesting to investigate whether the prevalence of *T. gondii* infection in TBI patients is similar to that reported in the general public.

## Conclusions

The findings of this study provide novel information pertaining to two common and often overlapping clinical conditions. The results demonstrate that a pre-existing *T. gondii* infection can alter the pathophysiological aftermath of TBI, including the neuroimmunological response, in both male and female mice. Furthermore, our MRI findings show that a pre-existing *T. gondii* infection exacerbates chronic brain damage after TBI, and this was particularly prominent in male mice. These findings indicate that a pre-existing *T. gondii* infection can modify TBI pathobiology in mice, provides important insights into potential sex differences, and, if validated in human studies, could be an important consideration in the clinical care of TBI patients. For example, if a TBI patient tests positive for *T. gondii* upon presentation to the clinic, and it is known that *T. gondii* infection can influence TBI pathobiology and recovery in a particular manner, then this could inform clinical decisions related to interventions and prognosis.

### Supplementary Information

**Additional file 1.** Supplementary tables including statistical details related to gene expression analysis in the ipsilateral cortex at 2-hours, 24-hours, 1-week, and 18-weeks post-injury.

**Additional file 2.** Supplementary figures of additional behavioral measures which depict limited differences between groups throughout each assessment.

## Data Availability

The dataset supporting the conclusions of this article are included within the article and its additional files.

## Abbreviations

3CT: 3-Chamber social test
ANOVA: Analysis of variance
BBB: Blood–brain barrier
CCI: Controlled cortical impact
CNS: Central nervous system
DPBS: Dulbecco’s phosphate buffered saline
DWI: Diffusion-weighted imaging
FOV: Field of view
FEW: Family wise error
GABA: Gamma-aminobutyric acid
IFC: Integrated fluidic circuit
i.p.: Intraperitoneal
MRI: Magnetic resonance imaging
PBS: Phosphate buffered saline
PFA: Paraformaldehyde
Pru: Prugniaud
RPM: Rotations per minute
s.c.: Subcutaneous
TBI: Traumatic brain injury
TE: Echo times
TR: Repetition time
