# Supplementary material for: A pre-existing Toxoplasma gondii infection exacerbates the pathophysiological response and extent of brain damage after traumatic brain injury in mice
Source: J Neuroinflammation. 2024 Jan 9;21:14. doi: 10.1186/s12974-024-03014-w (PMC10775436; doi:10.1186/s12974-024-03014-w)
Supplement: Supplementary file 1 — Additional file 1. Supplementary tables including statistical details related to gene expression analysis in the ipsilateral cortex at 2-hours, 24-hours, 1-week, and 18-weeks post-injury. [file 12974_2024_3014_MOESM1_ESM.docx]

**Table S1.** **Statistics of gene analyses of ipsilateral cortex tissue, at 2h, 24h, and 1week following CCI/sham**. Two-way ANOVA findings (F and *p* values), in order: infection, injury, infection* injury; or Kruskal-Wallis test findings (H value).

| **Gene** | **2h, Male** | **2h, Female** | **24h, Male** | **24h, Female** | **1wk, Male** | **1wk, Female** |
| --- | --- | --- | --- | --- | --- | --- |
| **Neuro-inflammation** | | | | | | |
| *GFAP* | F_1,18_=44.06, *p*<0.001  F_1,18_=0.01, *p*=0.941  F_1,18_=0.03, *p*=0.876 | H(3)=18.01, *p*<0.001 | F_1,19_=0.07, *p*=0.794  F_1,19_=26.76, *p*<0.001  F_1,19_=0.60, *p*=0.448 | H(3)=16.91, *p*=0.001 | F_1,20_=0.40, *p*=0.537  F_1,20_=44.36, *p*<0.001  F_1,20_=1.21, *p*=0.285 | F_1,20_=1.04, *p*=0.320  F_1,20_=26.62, *p*<0.001  F_1,20_=1.12, *p*=0.303 |
| *CD45* | F_1,18_=52.73, *p*<0.001  F_1,18_=1.39, *p*=0.254  F_1,18_=0.49, *p*=0.494 | H(3)=17.95, *p*<0.001 | F_1,19_=99.12, *p*<0.001  F_1,19_=7.71, *p*=0.012  F_1,19_=1.58, *p*=0.225 | F_1,19_=46.29, *p*<0.001  F_1,19_=20.79, *p*<0.001  F_1,19_=0.13, *p*=0.719 | F_1,20_=18.48, *p*=0.000  F_1,20_=12.80, *p*=0.002  F_1,20_=0.05, *p*=0.834 | F_1,20_=24.86, *p*=0.000  F_1,20_=8.46, *p*=0.009  F_1,20_=1.53, *p*=0.231 |
| *CD86* | F_1,18_=46.76, *p*<0.001  F_1,18_=7.09, *p*=0.016  F_1,18_=1.64, *p*=0.216 | F_1,20_=104.68, *p*<0.001  F_1,20_=8.32, *p*=0.009  F_1,20_=0.22, *p*=0.647 | H(3)=18.76, *p*<0.001 | H(3)=17.35, *p*=0.001 | F_1,20_=19.36, *p*=0.000  F_1,20_=14.81, *p*=0.001  F_1,20_=0.12, *p*=0.734 | F_1,20_=22.43, *p*=0.000  F_1,20_=11.55, *p*=0.003  F_1,20_=0.42, *p*=0.526 |
| *CD206* | N/A | N/A | H(3)=6.33, *p*=0.097 | F_1,19_=7.47, *p*=0.013  F_1,19_=0.73, *p*=0.404  F_1,19_=0.42, *p*=0.524 | F_1,20_=0.41, *p*=0.527  F_1,20_=4.48, *p*=0.047  F_1,20_<0.01, *p*=0.964 | F_1,20_=3.40, *p*=0.080  F_1,20_=5.43, *p*=0.030  F_1,20_=0.19, *p*=0.665 |
| *TREM2* | F_1,18_=30.46, *p*<0.001  F_1,18_=0.05, *p*=0.835  F_1,18_=11.06, *p*=0.307 | H(3)=17.39, *p*<0.001 | F_1,19_=37.42, *p*<0.001  F_1,19_=3.09, *p*=0.095  F_1,19_=0.06, *p*=0.817 | F_1,19_=46.89, *p*=0.000  F_1,19_=5.54, *p*=0.029  F_1,19_=1.70, *p*=0.208 | F_1,20_=5.68, *p*=0.027  F_1,20_=79.21, *p*<0.001  F_1,20_=0.11, *p*=0.742 | F_1,20_=7.01, *p*=0.015  F_1,20_=36.10, *p*<0.001  F_1,20_=1.24, *p*=0.279 |
| *CCR5* | F_1,18_=34.88, *p*<0.001  F_1,18_=0.02, *p*=0.890  F_1,18_=0.26, *p*=0.619 | F_1,20_=103.18, *p*<0.001  F_1,20_=2.30, *p*=0.145  F_1,20_=3.57, *p*=0.074 | F_1,19_=32.05, *p*<0.001  F_1,19_=7.69, *p*=0.012  F_1,19_=0.01, *p*=0.929 | F_1,19_=41.40, *p*=0.000  F_1,19_=31.26, *p*=0.000  F_1,19_=0.04, *p*=0.850 | F_1,20_=10.44, *p*=0.004  F_1,20_=10.41, *p*=0.004  F_1,20_<0.01, *p*=0.978 | F_1,20_=14.25, *p*=0.001  F_1,20_=10.97, *p*=0.003  F_1,20_=0.41, *p*=0.531 |
| *IBA1* | H(3)=16.00, *p*=0.001 | H(3)=17.95, *p*<0.001 | F_1,19_=116.18, *p*<0.001  F_1,19_=6.30, *p*=0.021  F_1,19_=0.43, *p*=0.518 | H(3)=18.24, *p*<0.001 | F_1,20_=21.91, *p*<0.001  F_1,20_=17.78, *p*<0.001  F_1,20_=0.01, *p*=0.920 | F_1,20_=21.98, *p*=0.000  F_1,20_=19.05, *p*<0.001  F_1,20_=0.43, *p*=0.519 |
| *TMEM119* | F_1,18_=16.04, *p*<0.001  F_1,18_=8.02, *p*=0.011  F_1,18_=3.50, *p*=0.078 | F_1,20_=35.77, *p*<0.001  F_1,20_=2.17, *p*=0.157  F_1,20_=0.48, *p*=0.829 | F_1,19_=19.40, *p*<0.001  F_1,19_<0.01, *p*=0.955  F_1,19_=0.03, *p*=0.860 | H(3)=14.03, *p*=0.003 | F_1,20_=21.05, *p*<0.001  F_1,20_=20.49, *p*<0.001  F_1,20_=0.03, *p*=0.860 | F_1,20_=32.98, *p*=0.000  F_1,20_=16.05, *p*=0.001  F_1,20_=2.87, *p*=0.106 |
| *GATA3* | F_1,18_=32.27, *p*<0.001  F_1,18_=1.08, *p*=0.314  F_1,18_=0.62, *p*=0.441 | H(3)=18.25, *p*<0.001 | H(3)=16.82, *p*<0.001 | F_1,19_=129.92, *p*<0.001  F_1,19_=6.58, *p*=0.020  F_1,19_=0.02, *p*=0.890 | F_1,20_=27.79, *p*<0.001  F_1,20_=1.72, *p*=0.204  F_1,20_=0.95, *p*=0.342 | F_1,20_=46.65, *p*=0.000  F_1,20_=0.85, *p*=0.369  F_1,20_<0.01, *p*=0.967 |
| *CXCR3* | F_1,18_=76.68, *p*<0.001  F_1,18_=0.44, *p*=0.516  F_1,18_<0.01, *p*=0.981 | H(3)=17.63, *p*<0.001 | H(3)=16.71, *p*<0.001 | H(3)=17.23, *p*=0.001 | H(3)=16.99, *p*=0.001 | H(3)=18.25, *p*=0.000 |
| *STAT1* | H(3)=15.34, *p*=0.002 | H(3)=17.35, *p*<0.001 | H(3)=18.09, *p*<0.001 | H(3)=17.96, *p*<0.001 | F_1,20_=27.96, *p*=0.000  F_1,20_=7.25, *p*=0.014  F_1,20_=0.06, *p*=0.805 | F_1,20_=41.65, *p*=0.000  F_1,20_=6.17, *p*=0.022  F_1,20_=0.02, *p*=0.883 |
| *SOCS1* | H(3)=14.56, *p*=0.002 | H(3)=18.13, *p*<0.001 | F_1,19_=50.12, *p*<0.001  F_1,19_=1.85, *p*=0.190  F_1,19_=0.81, *p*=0.379 | H(3)=14.19, *p*=0.003 | H(3)=12.72, *p*=0.005 | H(3)=15.77, *p*=0.001 |
| *FOXP3* | H(3)=13.69, *p*=0.003 | H(3)=17.55, *p<*0.001 | H(3)=14.01, *p*=0.003 | F_1,19_=23.97, *p*<0.001  F_1,19_=5.09, *p*=0.036  F_1,19_=0.36, *p*=0.554 | H(3)=15.04, *p*=0.002 | F_1,20_=44.48, *p*=0.000  F_1,20_=0.34, *p*=0.565  F_1,20_=0.71, *p*=0.410 |
| *TSPO* | H(3)=13.87, *p*=0.003 | H(3)=17.34, *p*<0.001 | F_1,19_=19.68, *p*<0.001  F_1,19_=9.84, *p*=0.005  F_1,19_=1.01, *p*=0.327 | H(3)=14.05, *p*=0.003 | F_1,20_=7.22, *p*=0.014  F_1,20_=14.77, *p*=0.001  F_1,20_=1.20, *p*=0.287 | F_1,20_=17.22, *p*=0.000  F_1,20_=8.24, *p*=0.009  F_1,20_=1.35, *p*=0.259 |
| *NLRP3* | F_1,18_=4.39, *p*=0.051  F_1,18_=78.53, *p*<0.001  F_1,18_=2.84, *p*=0.109 | F_1,20_=36.88, *p*<0.001  F_1,20_=108.28, *p*<0.001  F_1,20_=3.99, *p*=0.059 | F_1,19_=52.88, *p*<0.001  F_1,19_=10.79, *p*=0.004  F_1,19_=1.01, *p*=0.329 | F_1,19_=24.24, *p*<0.001  F_1,19_=16.14, *p*=0.001  F_1,19_=0.60, *p*=0.450 | F_1,20_=14.80, *p*=0.001  F_1,20_=18.13, *p*<0.001  F_1,20_=0.13, *p*=0.726 | F_1,20_=19.23, *p*<0.001  F_1,20_=8.77, *p*=0.008  F_1,20_=1.20, *p*=0.287 |
| *IL1β* | F_1,18_=5.78, *p*=0.027  F_1,18_=25.52, *p*<0.001  F_1,18_=3.18, *p*=0.092 | F_1,20_=19.52, *p*<0.001  F_1,20_=44.18, *p*<0.001  F_1,20_=0.42, *p*=0.526 | F_1,19_=15.50, *p*<0.001  F_1,19_=4.56, *p*=0.046  F_1,19_=0.04, *p*=0.840 | F_1,19_=7.35, *p*=0.014  F_1,19_=14.97, *p*=0.001  F_1,19_=0.16, *p*=0.690 | F_1,20_=8.96, *p*=0.007  F_1,20_=2.70, *p*=0.116  F_1,20_=0.07, *p*=0.796 | F_1,20_=7.95, *p*=0.011  F_1,20_=0.76, *p*=0.394  F_1,20_=4.19, *p*=0.054 |
| *IL2* | H(3)=10.20, *p*=0.017 | H(3)=18.15, *p*<0.001 | H(3)=16.92, *p*<0.001 | H(3)=10.36, *p*=0.016 | H(3)=14.50, *p*=0.002 | H(3)=10.70, *p*=0.013 |
| *IL6* | H(3)=10.54, *p*=0.015 | F_1,20_=14.33, *p*=0.001  F_1,20_=52.55, *p*<0.001  F_1,20_<0.01, *p*=0.985 | F_1,19_=1.65, *p*=0.215  F_1,19_=22.31, *p*<0.001  F_1,19_<0.01, *p*=0.969 | F_1,19_=2.10, *p*=0.164  F_1,19_=28.65, *p*=0.000  F_1,19_=0.12, *p*=0.737 | F_1,20_=6.87, *p*=0.016  F_1,20_=2.69, *p*=0.117  F_1,20_=0.10, *p*=0.750 | H(3)=9.89, *p*=0.020 |
| *IL10* | H(3)=13.44, *p*=0.004 | F_1,20_=62.17, *p*<0.001  F_1,20_=19.15, *p*<0.001  F_1,20_=1.30, *p*=0.269 | F_1,19_=130.20, *p*<0.001  F_1,19_=4.26, *p*=0.053  F_1,19_=0.23, *p*=0.637 | H(3)=14.79, *p*=0.002 | F_1,20_=26.34, *p*<0.001  F_1,20_=1.07, *p*=0.313  F_1,20_=0.62, *p*=0.441 | F_1,20_=16.54, *p*=0.001  F_1,20_=0.40, *p*=0.537  F_1,20_=1.14, *p*=0.715 |
| *IL12p40* | H(3)=16.54, *p*<0.001 | H(3)=19.45, *p*<0.001 | H(3)=17.26, *p*<0.001 | F_1,19_=54.74, *p*=0.000  F_1,19_=2.56, *p*=0.126  F_1,19_=0.72, *p*=0.406 | F_1,20_=29.32, *p*=0.000  F_1,20_=2.78, *p*=0.111  F_1,20_=0.38, *p*=0.546 | F_1,20_=35.49, *p*=0.000  F_1,20_=0.26, *p*=0.618  F_1,20_=1.93, *p*=0.180 |
| *IL33* | F_1,18_=2.56, *p*=0.127  F_1,18_=0.01, *p*=0.916  F_1,18_=0.02, *p*=0.893 | F_1,20_=7.55, *p*=0.012  F_1,20_=3.32, *p*=0.084  F_1,20_=1.03, *p*=0.322 | H(3)=4.65, *p*=0.200 | F_1,19_=7.89, *p*=0.011  F_1,19_=55.36, *p*=0.000  F_1,19_=2.76, *p*=0.113 | F_1,20_=0.11, *p*=0.746  F_1,20_=5.60, *p*=0.028  F_1,20_=1.04, *p*=0.319 | F_1,20_=0.55, *p*=0.465  F_1,20_=5.13, *p*=0.035  F_1,20_=0.08, *p*=0.780 |
| *TNFα* | H(3)=16.13, *p*=0.001 | H(3)=18.82, *p*<0.001 | F_1,19_=50.38, *p*<0.001  F_1,19_=14.95, *p*=0.001  F_1,19_=0.01, *p*=0.936 | F_1,19_=26.09, *p*<0.001  F_1,19_=30.18, *p*<0.001  F_1,19_=1.33, *p*=0.263 | F_1,20_=17.28, *p*=0.000  F_1,20_=7.29, *p*=0.014  F_1,20_=0.03, *p*=0.856 | F_1,20_=21.96, *p*=0.000  F_1,20_=4.52, *p*=0.046  F_1,20_=3.83, *p*=0.065 |
| *CSF1/*  *M-CSF* | F_1,18_=4.54, *p*=0.047  F_1,18_=3.08, *p*=0.096  F_1,18_=0.01, *p*=0.940 | F_1,20_=63.14, *p*<0.001  F_1,20_=8.99, *p*=0.007  F_1,20_=0.92, *p*=0.349 | F_1,19_=12.43, *p*=0.002  F_1,19_=6.76, *p*=0.018  F_1,19_=0.33, *p*=0.574 | F_1,19_=9.69, *p*=0.006  F_1,19_=12.58, *p*=0.002  F_1,19_=0.10, *p*=0.752 | F_1,20_=4.80, *p*=0.041  F_1,20_=21.44, *p*<0.001  F_1,20_=1.77, *p*=0.198 | F_1,20_=11.40, *p*=0.003  F_1,20_=15.04, *p*=0.001  F_1,20_=1.04, *p*=0.321 |
| *CSF2/*  *GM-CSF* | F_1,18_=9.38, *p*=0.007  F_1,18_=13.52, *p*=0.002  F_1,18_=2.02, *p*=0.172 | F_1,20_=66.82, *p*<0.001  F_1,20_=17.08, *p*<0.001  F_1,20_=0.34, *p*=0.565 | F_1,19_=52.20, *p*<0.001  F_1,19_=4.07, *p*=0.058  F_1,19_=2.17, *p*=0.157 | F_1,19_=22.82, *p*<0.001  F_1,19_=3.37, *p*=0.082  F_1,19_=0.14, *p*=0.714 | F_1,20_=10.86, *p*=0.004  F_1,20_=3.76, *p*=0.067  F_1,20_=0.08, *p*=0.784 | F_1,20_=25.66, *p*=0.000  F_1,20_=1.51, *p*=0.234  F_1,20_=4.14, *p*=0.055 |
| *CXCL10* | F_1,18_=48.55, *p*<0.001  F_1,18_=10.98, *p*=0.004  F_1,18_=4.91, *p*=0.040 | F_1,20_=206.00, *p*<0.001  F_1,20_=23.61, *p*<0.001  F_1,20_=4.94, *p*=0.038 | F_1,19_=141.73, *p*<0.001  F_1,19_=10.79, *p*=0.004  F_1,19_=0.25, *p*=0.625 | F_1,19_=66.57, *p*=0.000  F_1,19_=13.29, *p*=0.002  F_1,19_=0.07, *p*=0.794 | F_1,20_=12.59, *p*=0.002  F_1,20_=11.87, *p*=0.003  F_1,20_=0.01, *p*=0.936 | F_1,20_=29.78, *p*=0.000  F_1,20_=6.15, *p*=0.022  F_1,20_=4.21, *p*=0.054 |
| *IFNγ* | H(3)=18.51, *p*<0.001 | H(3)=18.52, *p*<0.001 | H(3)=17.85, *p*<0.001 | H(3)=16.82, *p*=0.001 | H(3)=17.06, *p*=0.001 | H(3)=17.37, *p*=0.001 |
| *TGFβ/*  *TGIF1* | F_1,18_=15.65, *p*<0.001  F_1,18_=16.65, *p*<0.001  F_1,18_=2.96, *p*=0.103 | F_1,20_=66.01, *p*<0.001  F_1,20_=24.01, *p*<0.001  F_1,20_<0.01, *p*=0.946 | H(3)=12.77, *p*=0.005 | F_1,19_=12.28, *p*=0.002  F_1,19_=29.10, *p*=0.000  F_1,19_=0.10, *p*=0.760 | F_1,20_=11.27, *p*=0.003  F_1,20_=19.94, *p*<0.001  F_1,20_=0.19, *p*=0.667 | F_1,20_=11.50, *p*=0.003  F_1,20_=14.73, *p*=0.001  F_1,20_=1.37, *p*=0.550 |
| *ARG1* | H(3)=2.20, *p*=0.531 | H(3)=11.45, *p*=0.010 | F_1,19_=2.72, *p*=0.116  F_1,19_=63.73, *p*<0.001  F_1,19_=3.38, *p*=0.082 | H(3)=16.62, *p*=0.001 | F_1,20_=1.04, *p*=0.319  F_1,20_=4.91, *p*=0.038  F_1,20_=0.37, *p*=0.552 | F_1,20_=1.20, *p*=0.287  F_1,20_=8.40, *p*=0.009  F_1,20_=0.07, *p*=0.800 |
| *CCL2* | H(3)=11.07, *p*=0.011 | H(3)=16.42, *p*<0.001 | F_1,19_=22.93, *p*<0.001  F_1,19_=30.03, *p*<0.001  F_1,19_=0.67, *p*=0.425 | F_1,19_=16.40, *p*=0.001  F_1,19_=30.42, *p*<0.001  F_1,19_=0.50, *p*=0.490 | F_1,20_=12.65, *p*=0.002  F_1,20_=8.97, *p*=0.007  F_1,20_=0.16, *p*=0.696 | F_1,20_=19.52, *p*=0.000  F_1,20_=4.87, *p*=0.039  F_1,20_=1.99, *p*=0.174 |
| *CCL4* | H(3)=14.04, *p*=0.003 | H(3)=17.77, *p*<0.001 | F_1,19_=7.10, *p*=0.015  F_1,19_=35.22, *p*<0.001  F_1,19_=1.19, *p*=0.289 | H(3)=15.19, *p*=0.002 | F_1,20_=0.78, *p*=0.389  F_1,20_=19.07, *p*<0.001  F_1,20_=0.05, *p*=0.825 | F_1,20_=3.35, *p*=0.082  F_1,20_=8.82, *p*=0.008  F_1,20_=5.19, *p*=0.034 |
| *CCL5* | F_1,18_=96.21, *p*<0.001  F_1,18_=5.04, *p*=0.038  F_1,18_=1.98, *p*=0.176 | H(3)=20.11, *p*<0.001 | H(3)=18.32, *p*<0.001 | H(3)=18.43, *p*<0.001 | F_1,20_=32.66, *p*<0.001  F_1,20_=8.42, *p*=0.009  F_1,20_=0.08, *p*=0.785 | F_1,20_=38.18, *p*=0.000  F_1,20_=2.92, *p*=0.103  F_1,20_=2.42, *p*=0.135 |
| *CCL12* | F_1,18_=46.93, *p*<0.001  F_1,18_=20.78, *p*<0.001  F_1,18_=3.71, *p*=0.070 | F_1,20_=152.94, *p*<0.001  F_1,20_=26.71, *p*<0.001  F_1,20_=5.64, *p*=0.028 | H(3)=10.98, *p*=0.012 | F_1,19_=11.86, *p*=0.003  F_1,19_=15.92, *p*=0.001  F_1,19_=0.92, *p*=0.350 | F_1,20_=12.53, *p*=0.002  F_1,20_=11.66, *p*=0.003  F_1,20_<0.01, *p*=0.950 | F_1,20_=24.17, *p*=0.000  F_1,20_=10.62, *p*=0.004  F_1,20_=2.29, *p*=0.146 |
| **Neuronal cell markers** | | | | | | |
| *MAP2* | F_1,18_=0.08, *p*=0.782  F_1,18_=0.01, *p*=0.921  F_1,18_=0.96, *p*=0.340 | F_1,20_=14.47, *p*=0.001  F_1,20_=0.77, *p*=0.391  F_1,20_=0.32, *p*=0.581 | H(3)=10.66, *p*=0.014 | H(3)=11.03, *p*=0.012 | F_1,20_=1.47, *p*=0.240  F_1,20_=4.28, *p*=0.052  F_1,20_=0.26, *p*=0.619 | F_1,20_=3.40, *p*=0.080  F_1,20_=4.37, *p*=0.050  F_1,20_=0.18, *p*=0.675 |
| **Glutamate pathway** | | | | | | |
| *GLUL* | F_1,18_=0.07, *p*=0.795  F_1,18_<0.01, *p*=0.964  F_1,18_=0.48, *p*=0.497 | F_1,20_=8.18, *p*=0.010  F_1,20_=4.60, *p*=0.044  F_1,20_=2.52, *p*=0.128 | F_1,19_=8.05, *p*=0.011  F_1,19_=2.22, *p*=0.152  F_1,19_=9.39, *p*=0.006 | F_1,19_=0.16, *p*=0.693  F_1,19_=14.83, *p*=0.001  F_1,19_=1.35, *p*=0.260 | F_1,20_=0.43, *p*=0.518  F_1,20_=1.61, *p*=0.219  F_1,20_=0.07, *p*=0.799 | F_1,20_=2.76, *p*=0.112  F_1,20_=7.96, *p*=0.011  F_1,20_=0.38, *p*=0.545 |
| *GRIN2B* | F_1,18_=0.46, *p*=0.505  F_1,18_=0.28, *p*=0.603  F_1,18_<0.01, *p*=0.979 | F_1,20_=6.05, *p*=0.023  F_1,20_=5.76, *p*=0.026  F_1,20_<0.01, *p*=0.975 | F_1,19_=0.03, *p*=0.866  F_1,19_=0.22, *p*=0.642  F_1,19_=9.91, *p*=0.005 | H(3)=6.39, *p*=0.094 | F_1,20_=0.55, *p*=0.466  F_1,20_=0.49, *p*=0.494  F_1,20_=0.02, *p*=0.893 | F_1,20_=1.35, *p*=0.259  F_1,20_=1.23, *p*=0.282  F_1,20_<0.01, *p*=0.948 |
| *GLT1* | F_1,18_=4.06, *p*=0.059  F_1,18_=0.04, *p*=0.836  F_1,18_=0.72, *p*=0.407 | F_1,20_=6.75, *p*=0.017  F_1,20_=0.59, *p*=0.452  F_1,20_=1.43, *p*=0.245 | F_1,19_=0.69, *p*=0.417  F_1,19_=2.90, *p*=0.105  F_1,19_=3.86, *p*=0.064 | H(3)=13.46, *p*=0.004 | F_1,20_=5.26, *p*=0.033  F_1,20_=1.73, *p*=0.204  F_1,20_=0.19, *p*=0.666 | F_1,20_=4.11, *p*=0.056  F_1,20_=5.49, *p*=0.030  F_1,20_=1.19, *p*=0.289 |
| *GLAST* | F_1,18_=1.87, *p*=0.188  F_1,18_=0.21, *p*=0.653  F_1,18_=2.81, *p*=0.111 | F_1,20_=13.32, *p*=0.002  F_1,20_=0.17, *p*=0.685  F_1,20_<0.01, *p*=0.965 | F_1,19_=12.60, *p*=0.002  F_1,19_=0.33, *p*=0.571  F_1,19_=1.82, *p*=0.193 | F_1,19_=10.56, *p*=0.004  F_1,19_=0.27, *p*=0.609  F_1,19_=0.29, *p*=0.594 | F_1,20_=3.07, *p*=0.095  F_1,20_=22.24, *p*<0.001  F_1,20_=1.44, *p*=0.244 | F_1,20_=5.03, *p*=0.036  F_1,20_=11.81, *p*=0.003  F_1,20_=0.00, *p*=0.988 |
| *GAD1* | F_1,18_=0.31, *p*=0.586  F_1,18_=0.43, *p*=0.521  F_1,18_=1.41, *p*=0.711 | F_1,20_=0.84, *p*=0.369  F_1,20_=1.78, *p*=0.197  F_1,20_=0.66, *p*=0.425 | F_1,19_=6.81, *p*=0.017  F_1,19_=4.68, *p*=0.043  F_1,19_=0.01, *p*=0.946 | H(3)=4.50, *p*=0.212 | F_1,20_=1.70, *p*=0.207  F_1,20_=18.59, *p*<0.001  F_1,20_=0.61, *p*=0.443 | H(3)=4.49, *p*=0.213 |
| *PHGDH* | F_1,18_=1.04, *p*=0.321  F_1,18_=0.02, *p*=0.897  F_1,18_=2.08, *p*=0.166 | F_1,20_<0.01, *p*=0.932  F_1,20_=0.02, *p*=0.900  F_1,20_=1.47, *p*=0.240 | F_1,19_=0.82, *p*=0.376  F_1,19_=0.76, *p*=0.393  F_1,19_=1.64, *p*=0.216 | F_1,19_=0.96, *p*=0.338  F_1,19_=4.85, *p*=0.040  F_1,19_=2.68, *p*=0.118 | F_1,20_=0.15, *p*=0.701  F_1,20_=10.21, *p*=0.005  F_1,20_=2.22, *p*=0.152 | F_1,20_=0.31, *p*=0.582  F_1,20_=21.35, *p*<0.001  F_1,20_=0.00, *p*=0.983 |
| *PPARγ* | F_1,18_=0.22, *p*=0.645  F_1,18_=0.07, *p*=0.795  F_1,18_=0.48, *p*=0.497 | F_1,20_=0.68, *p*=0.418  F_1,20_=6.34, *p*=0.020  F_1,20_=2.35, *p*=0.141 | F_1,19_=0.74, *p*=0.401  F_1,19_=9.37, *p*=0.006  F_1,19_=0.20, *p*=0.660 | F_1,19_=2.02, *p*=0.171  F_1,19_=6.12, *p*=0.023  F_1,19_=0.98, *p*=0.335 | F_1,20_=0.02, *p*=0.892  F_1,20_=17.03, *p*=0.001  F_1,20_=1.59, *p*=0.222 | F_1,20_=2.28, *p*=0.147  F_1,20_=1.43, *p*=0.246  F_1,20_=0.02, *p*=0.901 |
| **Oxidative stress** | | | | | | |
| *IDO1* | H(3)=17.15, *p*<0.001 | H(3)=17.31, *p*<0.001 | H(3)=17.26, *p*<0.001 | H(3)=17.68, *p*=0.001 | H(3)=15.86, *p*=0.001 | H(3)=17.81, *p*=0.000 |
| *NOS2* | H(3)=6.224, *p*=0.101 | H(3)=17.41, *p*<0.001 | H(3)=18.42, *p*<0.001 | H(3)=11.14, *p*=0.011 | H(3)=12.54, *p*=0.006 | H(3)=9.10, *p*=0.028 |
| *CYBB* | F_1,18_=0.07, *p*=0.795  F_1,18_<0.01, *p*=0.964  F_1,18_=0.48, *p*=0.497 | H(3)=17.97, *p*<0.001 | H(3)=18.32, *p*<0.001 | H(3)=19.04, *p*<0.001 | F_1,20_=27.05, *p*<0.001  F_1,20_=15.98, *p*=0.001  F_1,20_=0.11, *p*=0.746 | F_1,20_=29.35, *p*=0.000  F_1,20_=11.15, *p*=0.003  F_1,20_=1.73, *p*=0.204 |

**Table S2.** **Statistics of gene analyses of ipsilateral cortex tissue, at 18 weeks following CCI/sham**. Two-way ANOVA findings (F and *p* values), in order: infection, injury, infection* injury; or Kruskal-Wallis test findings (H value).

| **Gene** | **18wk, Male** | **18wk, Female** |
| --- | --- | --- |
| **BBB integrity/vascular health** | | |
| *ICAM1* | F_1,15_=24.38, *p*=0.000  F_1,15_=0.15, *p*=0.705  F_1,15_=5.77, *p*=0.030 | H(3)=14.43, *p*=0.002 |
| *OCLN* | F_1,15_<0.01, *p*=0.940  F_1,15_=5.42, *p*=0.034  F_1,15_=2.90, *p*=0.109 | F_1,15_=0.04, *p*=0.843  F_1,15_=0.04, *p*=0.837  F_1,15_=0.36, *p*=0.555 |
| *TJP1* | F_1,15_=2.82, *p*=0.114  F_1,15_=5.93, *p*=0.028  F_1,15_=0.08, *p*=0.783 | F_1,15_=1.25, *p*=0.280  F_1,15_=0.60, *p*=0.450  F_1,15_=1.43, *p*=0.250 |
| *VEGFA* | H(3)=6.95, *p*=0.074 | F_1,15_=12.22, *p*=0.003  F_1,15_=2.13, *p*=0.16  F_1,15_=0.68, *p*=0.422 |
| *SYNAPTOPHYSIN* | H(3)=6.95, *p*=0.074 | F_1,15_=5.25, *p*=0.037  F_1,15_=1.39, *p*=0.256  F_1,15_=1.84, *p*=0.195 |
| *HIF1A* | F_1,15_=0.06, *p*=0.803  F_1,15_=0.71, *p*=0.413  F_1,15_=2.74, *p*=0.119 | F_1,15_=1.86, *p*=0.192  F_1,15_=0.67, *p*=0.428  F_1,15_=0.01, *p*=0.922 |
| **Neuronal cell markers** | | |
| *MAP2* | F_1,15_=2.36, *p*=0.145  F_1,15_=0.35, *p*=0.563  F_1,15_=2.44, *p*=0.139 | F_1,15_=14.31, *p*=0.002  F_1,15_=8.63, *p*=0.010  F_1,15_=9.74, *p*=0.007 |
