# Supplementary material for: A pre-existing Toxoplasma gondii infection exacerbates the pathophysiological response and extent of brain damage after traumatic brain injury in mice
Source: J Neuroinflammation. 2024 Jan 9;21:14. doi: 10.1186/s12974-024-03014-w (PMC10775436; doi:10.1186/s12974-024-03014-w)
Supplement: Supplementary file 2 — Additional file 2. Supplementary figures of additional behavioral measures which depict limited differences between groups throughout each assessment. [file 12974_2024_3014_MOESM2_ESM.docx]

**
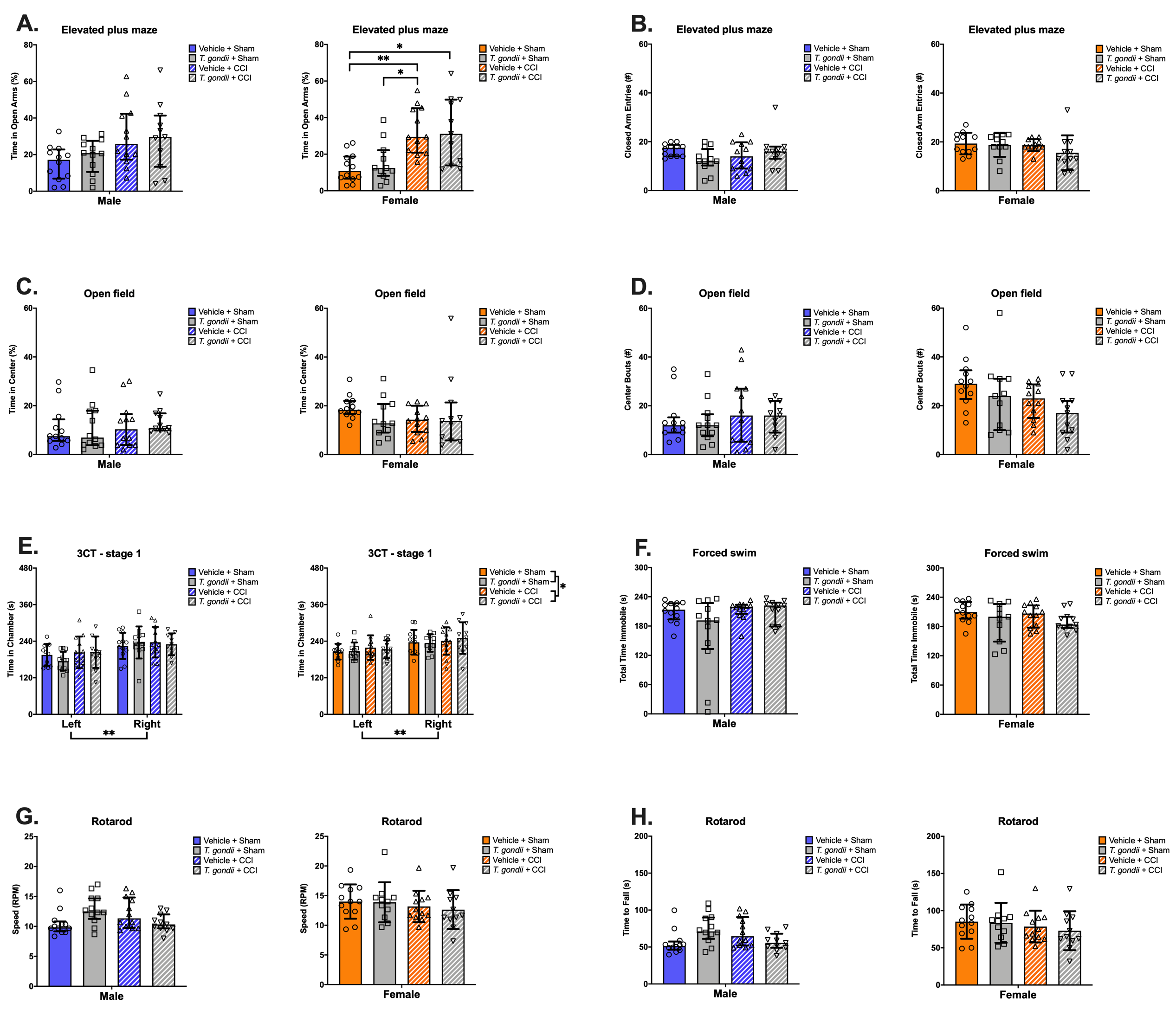
**

**Figure S1. Behavioral testing revealed decreased anxiety in female CCI mice.** Vehicle + CCI and *T. gondii* + CCI females spent more time in the open arms of the elevated plus maze compared to Vehicle + Sham females **(A)**, and no differences were seen between male groups. Male and female mice, irrespective of group, made a similar number of entries into the closed arms of the elevated plus maze **(B)**. Similarly, males and females, regardless of their group, spent a comparable amount of time in the center of the open field **(C)** and no differences were observed on the number of center bouts **(D)**. In stage 1 of the 3-chamber test, both male and female mice had a preference for the right chamber compared to the left, and a main effect of injury was found in female mice **(E)**. No differences between male and female groups were detected in the forced swim **(F)**. Differences in the average speed and time to fall on Rotarod were found between male mice groups, yet multiple comparisons did not reach significance (**G, H)**. Female mice performed equally on the Rotarod. Data displayed as mean ± SD or as median with interquartile range, where appropriate. * *p<* 0.05, ** *p* ≤ 0.01.


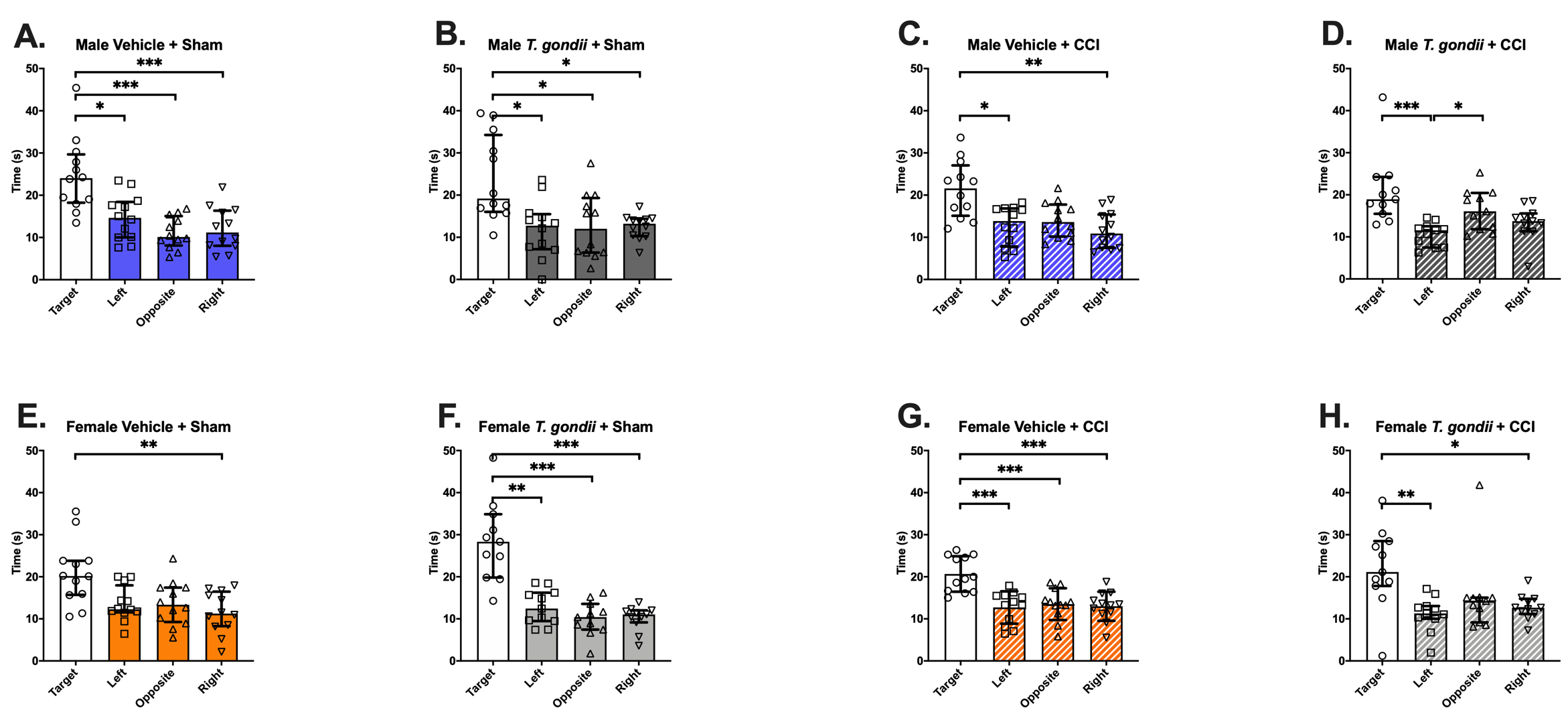


**Figure S2. Quadrant preference in the Morris water maze probe test varied across male and female groups.** Male Vehicle + Sham **(A)** and male *T. gondii* + Sham **(B)** mice had a singular preference for the target platform quadrant, yet male Vehicle + CCI mice spent longer in the target quadrant compared to only the left and right quadrants **(C)**. Male *T. gondii* + CCI mice uniquely spent more time in the target and opposite quadrants compared to the left quadrant, although no differences were observed between the time spent in the target quadrant compared to the opposite nor right quadrant **(D)**. Female Vehicle + Sham mice spent more time in the target quadrant compared to the right quadrant **(E)**, and both female *T. gondii* + Sham and Vehicle + CCI mice had a singular preference for the target quadrant **(F, G)**. Alike the male Vehicle + CCI group, female *T. gondii* + CCI mice spent longer in the target quadrant compared to the left and right quadrants **(H)**. Data displayed as mean ± SD or as median with interquartile range, where appropriate. * *p<* 0.05, ** *p* ≤ 0.01, *** *p* ≤ 0.001.


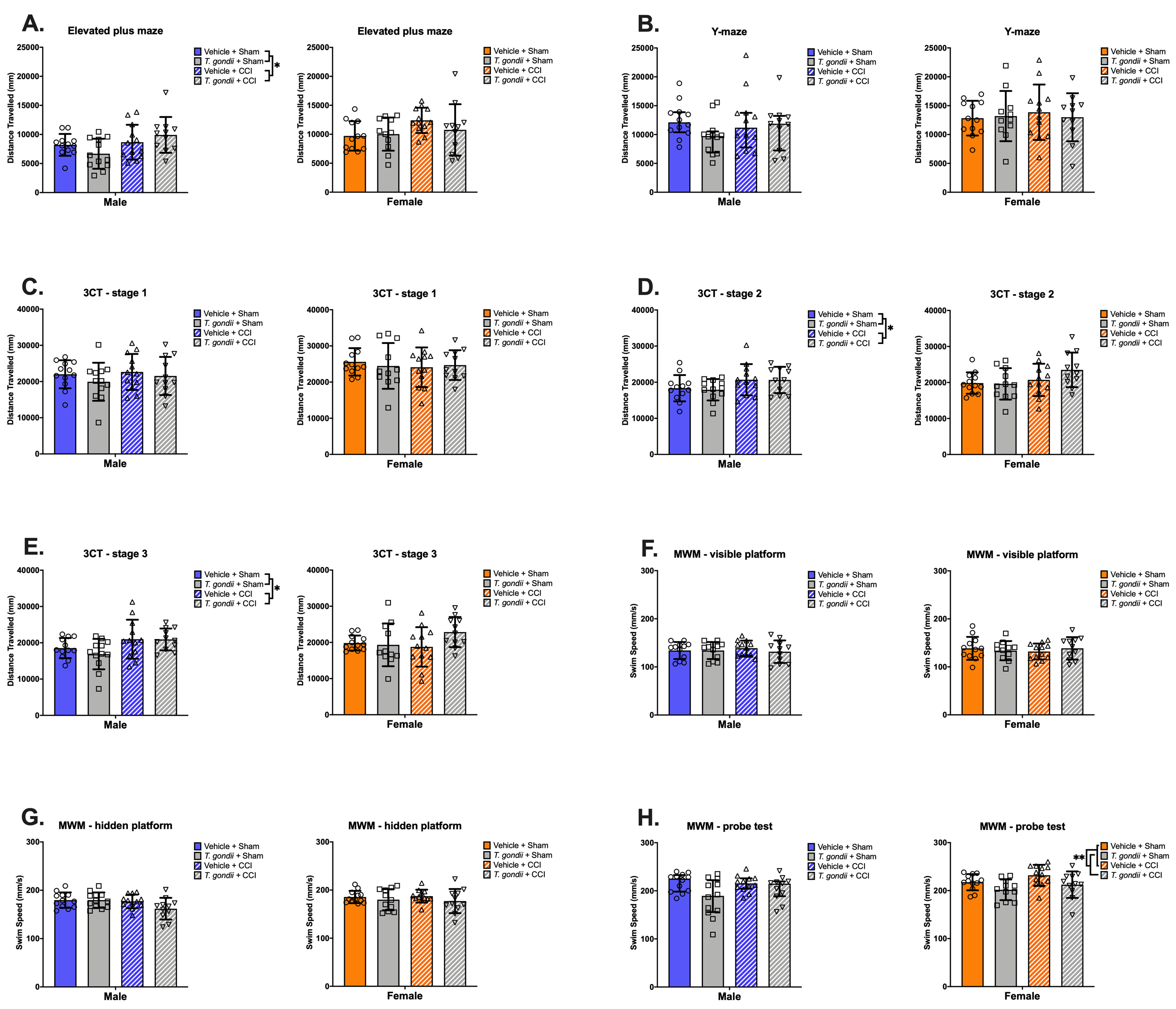


**Figure S3. Limited differences in the general activity of mice were found through behavior testing.** Male CCI mice travelled more throughout the elevated plus maze **(A)**, and no differences were seen between female groups. No differences in distance travelled in Y-maze were observed between male nor female groups **(B)**. Likewise, male and female groups in stage 1 of the 3-chamber test had similar activity levels **(C)**. However, male CCI mice travelled more than sham-injured mice in stage 2 and stage 3 of the 3-chamber test **(D, E**). No differences between female groups were seen in either stage 2 or stage 3. In the Morris water maze, no differences in swim speed were observed during the visible platform **(F)** or hidden platform stage **(G)**. *T. gondii* female mice had a decreased swim speed in the probe test compared to Vehicle mice, yet no group differences were detected in male mice **(H)**. Data displayed as mean ± SD or as median with interquartile range, where appropriate. * *p<* 0.05, ** *p* ≤ 0.01.
